# Half a Century of Per‐ and Polyfluoroalkyl Substances in Northern Gannet Eggs: Impact of Regulations

**DOI:** 10.1002/jat.70175

**Published:** 2026-03-27

**Authors:** Anaïs Fournier, Anaïs Kerric, Raphaël A. Lavoie

**Affiliations:** ^1^ Department of Chemistry University of Bordeaux Talence France; ^2^ Science and Technology Branch, Environment and Climate Change Canada Québec Québec Canada

**Keywords:** egg, long‐term trends, northern gannet, PFAS, regulations

## Abstract

Per‐ and polyfluoroalkyl substances (PFAS), known as “forever chemicals”, are persistent organic pollutants that have been widely used in the industry across the globe for many decades. Their chemical stability, bioaccumulation, and toxicity pose major environmental and health risks. This study examines temporal trends in the concentrations of 17 PFASs in northern gannet ( *Morus bassanus* ) eggs from the Gulf of St. Lawrence, Canada, over a 55‐year period (1969–2024). Analyses include 13 perfluoroalkyl carboxylic acids (PFCAs) and four perfluoroalkyl sulfonic acids (PFSAs). Stable isotopes of carbon (δ^13^C) and nitrogen (δ^15^N), lipid content, and moisture content were analyzed. Generalized additive models (GAMs), adjusted for lipids and for both lipids and δ^15^N, were applied to assess how chemical properties, industrial use, regulatory actions, and trophic dynamics influence PFAS trends. Perfluorooctanesulfonic acid (PFOS) was the dominant compound throughout the study period, peaking in the mid‐1990s and declining thereafter due to regulatory measures and voluntary phase‐outs. However, PFOS and other PFASs show high persistence, with estimated ecological half‐lives for ∑_17_PFASs of 60 years with lipid correction and 36 years after lipid and δ^15^N correction. Some long‐chain PFCAs, such as perfluorododecanoic acid (PFDoA; 12 carbons) and perfluorotridecanoic acid (PFTrDA; 13 carbons), have shown a delayed response to regulations. Moreover, PFOS and PFOS + perfluorohexanesulfonic acid (PFHxS) concentrations in northern gannet eggs remained above established toxicological thresholds for 30 years, indicating ecotoxicological risks for marine top predators. These findings highlight the persistent nature of PFAS contamination and underline the importance of regulations, long‐term research, and risk assessment.

## Abstract 2

Temporal trends (1969–2024) in concentrations of 17 per‐ and polyfluoroalkyl substances (PFASs) in northern gannet ( *Morus bassanus* ) eggs from the Gulf of St. Lawrence, Canada, were analyzed. The profile was dominated by perfluorooctanesulfonic acid (PFOS), peaking in the mid‐1990s and declining after phase‐out actions. Some long‐chain perfluoroalkyl carboxylic acids (PFCAs) declined more slowly. Ecological half‐lives of ∑_17_PFASs were estimated between 36 and 60 years. PFOS and PFOS + perfluorohexanesulfonic acid (PFHxS) levels exceeded toxicological thresholds for decades, indicating persistent risk.

## 1 Introduction

Per‐ and polyfluoroalkyl substances (PFAS), developed in the 1950s, are widely used in industrial and commercial applications, including in nonstick coatings, fire‐fighting foams, and water‐resistant textiles (Gaines 2023). Their structure, characterized by strong carbon‐fluorine bonds, confers exceptional stability, limiting their environmental degradation and earning them the nickname “forever chemicals” (Abunada et al. 2020; Ankley et al. 2020). PFAS are amphiphilic, with a hydrophobic fluorinated carbon chain and a hydrophilic head. Their behavior varies depending on carbon chain length and functional group, which influence their persistence, mobility, and bioaccumulation (Ohoro et al. 2024).

The most studied subgroups are perfluoroalkyl carboxylic acids (PFCA) and sulfonic acids (PFSA; Buck et al. 2011). PFSA contain a sulfonate group (–SO_3_^−^) attached to a perfluorinated carbon chain. This group promotes interactions with proteins and enhances its sorption to particles and sediments. The negatively charged sulfonate group can bind to positively charged sites, such as those on albumin, facilitating PFSA transport within the organism and accumulation in specific tissues (Forsthuber et al. 2020; Ohoro et al. 2024). PFCA, which contain a carboxylate group (–COO^−^), exhibits greater polarity. They disperse more readily in water, particularly when they have short carbon chains, which increases their mobility in aquatic environments (Abunada et al. 2020; Ohoro et al. 2024). The chain length also influences their environmental fate and toxicokinetics: long‐chain compounds (≥ 8 carbons [C8] for PFCA; ≥ C6 for PFSA) show a higher affinity for lipids due to their extended perfluorinated C–F chains, promoting their incorporation into plasma membranes and their accumulation in biological tissues (Ohoro et al. 2024).

PFAS have been detected in all ecosystems, including remote areas, demonstrating their long‐range transport and global ubiquity (Sun et al. 2023). Their widespread presence raises major environmental health concerns, with harmful effects observed in birds, including hormonal disruption, reproductive impairment, and weakened immune function (Ankley et al. 2020; Dennis et al. 2022; Kreitsberg et al. 2023; Humann‐Guilleminot et al. 2024; Ricolfi et al. 2024).

In response to environmental and health concerns, several national and international regulations have been established to restrict the use of PFAS. In Canada, a Risk Management Plan for perfluorooctanesulfonic acid (PFOS) was implemented and its manufacture, use, sale, and importation were banned in 2008 (Government of Canada 2006, 2025a). In 2016, this ban was extended to perfluorooctanoic acid (PFOA) and several long‐chain PFCAs (Government of Canada 2025b, 2025c). At the international level, PFOS was listed in Annex B of the Stockholm Convention in 2009, PFOA in Annex A in 2019, and perfluorohexanesulfonic acid (PFHxS) in Annex A in 2022 (United Nations Environment Programme (UNEP) 2009, 2019, 2022). However, these measures cover only a small fraction of the estimated 14,000 PFAS compounds (U.S. Environmental Protection Agency (EPA) 2023), leaving many unregulated and contributing to ongoing environmental release. Therefore, the overall impact of these restrictions on PFAS concentrations remains uncertain.

The Gulf of St. Lawrence (Canada), a highly ecologically and economically significant ecosystem, is particularly vulnerable to contamination by various chemicals, including PFAS. Fed by the Great Lakes and numerous rivers, the St. Lawrence River receives industrial and urban discharges from the United States and surrounding Canadian provinces (Xia et al. 2024). A recent assessment confirms widespread PFAS presence not only in the St. Lawrence River but also in its tributaries (Teymoorian et al. 2025). PFAS accumulate and biomagnify in aquatic food webs, leading to elevated concentrations in top predator fish, aquatic birds, and marine mammals, including harbor porpoises ( *Phocoena phocoena* ) and pinnipeds like gray seals ( *Halichoerus grypus* ) and harp seals ( *Pagophilus groenlandicus* ) (Ren et al. 2022; Khalid et al. 2024).

In this context, seabirds play a crucial role as sentinels of ecosystem health, as they occupy a high trophic level and therefore are particularly prone to bioaccumulation of contaminants (Gaspar et al. 2022). Among them, the northern gannet ( *Morus bassanus* ), a strictly piscivorous species, has been widely used to monitor contamination (Champoux et al. 2015, 2017; Pereira et al. 2021). Research on temporal trends of contaminants in the northern gannet population from Bonaventure Island, located in the Gulf of St. Lawrence, began in the 1960s. It was initiated in response to dichlorodiphenyldichloroethylene (DDE) egg contamination, a metabolite of dichlorodiphenyltrichloroethane (DDT). Exposure to this compound caused eggshell thinning, reduced reproductive success, and led to a population decline (Chapdelaine et al. 1987; Elliott et al. 1988). Strict regulation of DDT and other persistent organic pollutants (POPs) enabled population recovery in the 1970s (Chardine et al. 2013), paving the way for long‐term research on contaminant exposure and ecosystem health (Champoux et al. 2015, 2017). However, there are currently very limited data on PFAS contamination in northern gannets, despite their role as top predators and sentinel species of marine ecosystems. To address this knowledge gap, the analysis of their eggs provides an effective approach to assess contaminant transfer from females to offspring (Ricolfi et al. 2024), allowing reliable evaluation of temporal trends without significantly affecting population size.

This study aims to assess how the increasing use of PFAS over the past decades has influenced their concentrations in seabird eggs and to evaluate the effectiveness of changes in industrial practices and environmental regulations. Specifically, this study (1) analyzes temporal trends in PFAS concentrations in northern gannet eggs collected from 1969 to 2024; (2) evaluates the impact of regulatory actions on PFAS concentrations over time; and (3) estimates potential toxicological risks by comparing measured concentrations with established thresholds. It is hypothesized that PFAS targeted by regulations decline following their implementation.

## 2 Methods

### 2.1 Egg Collection

From 1969 to 2024, fresh northern gannet eggs were collected approximately every 5 years during early incubation (May–June) on Bonaventure Island (48°29′N, 64°09′W) part of the Parc National de l'Île‐Bonaventure‐et‐du‐Rocher‐Percé (Sépaq), the largest gannet colony in North America (50,000 estimated nests) and the second largest worldwide. In some years, sampling was increased to address specific research objectives (Table 1). On average, approximately 15 eggs were collected per year, depending on study needs and field conditions, from active nests. Eggs were kept cool in the field before transport to the National Wildlife Research Centre (NWRC, Ottawa, Ontario, Canada) for preparation and analysis. All collections followed Canadian Wildlife Service and Sépaq Park permits and guidelines, as well as ethical protocols from the Committee of Environment and Climate Change Canada.

**TABLE 1:** Mean values of moisture content (%), lipid content (%), stable nitrogen (δ^15^N) and carbon (δ^13^C) isotope ratios (‰), and PFAS^a^ concentrations or PFAS sums (ng/g wet weight) in northern gannet ( *Morus bassanus* ) eggs from Bonaventure Island, Québec.

Year	1969^b^	1973^b^	1974^b^	1976^b^	1984^b^	1989^b^	1994^c^	1999^c^	2004^c^	2009^c^	2012^c^	2014^c^	2019^c^	2024^d^
*n* (*n* eggs)	5	6	6	6	6	6	3 (5)	3 (5)	3 (5)	3 (5)	3 (5)	3 (5)	3 (5)	3 (5)
Moisture	83.6	84.3	83.8	84.0	83.6	82.8	82.6	83.7	82.3	83.7	84.7	83.0	84.0	84.3
Lipid	3.7	3.9	4.2	4.3	3.9	4.4	4.6	5.0	5.3	4.4	5.1	4.5	3.9	4.0
δ^15^N	15.1	14.2	NA	14.8	15.1	15.0	15.0	14.5	14.4	14.8	15.0	15.0	15.5	15.2
δ^13^C	−20.4	−20.9	NA	−20.5	−19.9	−19.8	−19.8	−19.8	−20.2	−20.0	−19.9	−19.4	−19.2	−21.5
PFOA	ND	0.097	0.128	0.238	0.223	0.299	0.665	0.847	0.783	0.485	0.417	0.333	0.359	0.497
PFNA	0.101	0.082	0.117	0.281	1.09	6.04	4.21	7.23	9.42	12.1	4.99	4.65	3.22	3.68
PFDA	0.333	0.298	1.13	0.593	1.62	3.02	2.50	3.63	4.53	5.83	4.45	5.36	4.20	3.71
PFUdA	0.770	0.709	0.814	1.14	4.64	8.46	7.03	12.3	16.4	18.1	10.6	10.9	7.31	6.39
PFDoA	0.338	0.302	0.342	0.474	1.81	2.46	2.23	3.25	4.17	4.25	2.94	2.98	2.37	1.76
PFTrDA	0.583	0.589	0.618	0.711	3.66	5.36	5.90	8.15	13.2	10.1	6.35	5.55	3.43	3.12
PFTeDA	0.17	0.191	0.201	0.230	1.00	1.35	1.50	2.40	2.81	2.14	1.67	1.43	1.18	1.03
PFHxS	ND	0.039	0.092	0.142	0.158	0.231	0.482	0.686	0.475	0.408	0.322	0.252	0.244	0.192
PFOS	25.5	14.7	16.7	38.5	47.3	67.3	76.1	99.9	54.8	57.2	53.9	62.6	37.8	26.8
PFDS	0.080	ND	ND	0.854	2.28	4.24	4.06	5.71	2.77	1.53	1.25	1.67	0.994	0.860
Σ_13_PFCAs	2.30	2.27	3.35	3.66	14.0	27.0	24.0	37.9	51.3	52.9	31.4	31.2	22.1	20.2
Σ_4_PFSAs	25.6	14.7	16.8	39.6	49.7	71.8	80.6	106.3	58.1	59.1	55.5	64.5	39.0	27.8
Σ_17_PFASs	27.9	17.0	20.2	43.1	63.8	98.8	104.6	144.2	109.4	112	86.9	95.7	61.1	48.0

Abbreviations: *n* (*n* eggs), the number of samples analyzed (pooled or individual egg; number before the parentheses) and the number of eggs per pool (number in parentheses); NA, not analyzed; ND, not detected; Σ_13_PFCAs, sum of PFBA, PFPeA, PFHxA, PFHpA, PFOA, PFNA, PFDA, PFUdA, PFDoA, PFTrDA, PFTeDA, PFHxDA, and PFODA; Σ_4_PFSAs, sum of PFBS, PFHxS, PFOS, and PFDS.

^a^ PFBA, PFPeA, PFHxA, PFHpA, PFHxDA, PFODA, and PFBS did not yield data or sufficient data above detection limits.

^b^ PFAS analyzed on individual eggs.

^c^ PFAS analyzed on 3 pools of 5 eggs.

^d^ PFAS analyzed on individual eggs that were pooled mathematically (3 means of 5 eggs).

### 2.2 Contaminant Analyses

Over the long‐term period of contaminant research in gannet eggs (1969–2024), different pooling scenarios were used depending on the samples available, which had to be analyzed either individually or pooled. From 1969 to 1989, five or six individual eggs were analyzed for PFAS per year. At that time, pooled analyses had not yet been implemented, so eggs were archived and measured individually. From 1994 onward, egg pools were analyzed, with three pools of five eggs per year. Pooling of eggs is a well‐established strategy in contaminant studies for wildlife and humans, reducing interindividual variability while providing a reliable estimate of the mean concentration (Turle and Collins 1992; Gewurtz et al. 2011; Heffernan et al. 2014). This approach was used by Kesic et al. (2023), in a long‐term study of PFAS concentrations in seabird eggs. It relies on the assumption that eggs included in each pool are randomly selected and that interindividual variability is reduced, ensuring that pooled measurements reflect annual population means. For 2024, individual eggs were analyzed for another study (unpublished data; *n* = 32). To maintain continuity with previous years analyzed using pooled samples, 15 of the least developed eggs were combined into three mathematical pools of five eggs each. This allowed the 2024 data to be directly comparable with earlier pooled measurements while preserving the statistical integrity of the temporal trend analysis (Table 1).

The archived egg samples from the National Wildlife Specimen Bank at NWRC for the 1969–2019 period were maintained at −40°C during that period and aliquots from bulk jars or individual samples were collected with chemically cleaned (triple rinse of acetone and hexane) stainless tools, following the procedures described by Champoux et al. (2015, 2017). The samples collected in 2024 were homogenized in the lab before being stored at −40°C until analysis. All samples were homogenized using a Polytron homogenizer or a ball mill, depending on the development of eggs. Although the samples span a broad temporal range (1969–2024), analyses were conducted in 2024. Analyses were performed using the same standardized analytical protocol in a Canadian Association for Laboratory Accreditation (CALA)‐accredited laboratory at NWRC. For contaminant extraction, 1.0 g of homogenized egg sample was taken, and an internal standard was added, followed by 10 mL of a 1% acetic acid solution in acetonitrile. After vortexing, agitation, and sonication, a salt mixture (magnesium sulfate and sodium acetate, 4:1 ratio) was added for extraction. The sample was then centrifuged, and the supernatant was transferred into a tube containing 50 mg of SupelClean ENVI‐18 for further purification. After agitation, centrifugation, and evaporation to dryness, the extract was reconstituted in a water/methanol mixture (25:75) and filtered using a 0.22‐μm nylon filter before being transferred into an autosampler vial.

Chromatographic analyses were performed on an Agilent 1260/90 HPLC system, using an X‐Terra MS C18 pre‐column and an Infinity Lab Poroshell 120 EC‐C18 analytical column. The column temperature was maintained at 50°C, and the sampler temperature was set to 25°C. The mobile phase consisted of a mixture of ammonium acetate in water (phase A) and in methanol (phase B), with a gradient to effectively separate the contaminants. The flow rate was 600 μL/min, and an injection volume of 2 μL was used. The analysis was completed by mass spectrometry (Sciex API 5500) in TurboSpray mode and negative polarity, with Multiple Reaction Monitoring (MRM) to detect the 17 PFASs and the nine associated IS. The spectrometer parameters were optimized to ensure sensitivity and precision of the measurements, with specific MRM transitions for each contaminant.

### 2.3 Quality Assurance and Quality Control

Standard solutions of PFAS and internal standards were supplied by Wellington Labs: a 17‐PFC mixture in methanol at 2000 ng/mL (containing perfluorobutanoic acid [PFBA], perfluoropentanoic acid [PFPeA], perfluorohexanoic acid [PFHxA], perfluoroheptanoic acid [PFHpA], PFOA, perfluorononanoic acid [PFNA], perfluorodecanoic acid [PFDA], perfluoroundecanoic acid [PFUdA], perfluorododecanoic acid [PFDoA], perfluorotridecanoic acid [PFTrDA], perfluorotetradecanoic acid [PFTeDA], perfluorohexadecanoic acid [PFHxDA], perfluorooctadecanoic acid [PFODA], perfluorobutanesulfonic acid [PFBS], PFHxS, PFOS, and perfluorodecanesulfonic acid [PFDS]) and an L‐PFOS solution at 50 μg/mL. Calibration curves were established at six concentration levels, ranging from 0.25 to 50 ng/mL for 16 of the PFCs and from 2.5 to 500 ng/mL for L‐PFOS, with linear regression (*R* > 0.995; weighting 1/x). Calculations were performed using peak areas and the internal standard method. Most compounds were monitored with two transitions, but only the primary fragment was used for quantification. Sulfonated compounds like PFBS, PFHxS, and PFDS were reported as “L” isomers (linear).

Three to five blanks (water/methanol, 25:75) were injected to monitor cross‐contamination during injection. The results of these blanks were checked but are not reported. A bio‐organic egg homogenate, spiked with the internal standard solution, was analyzed alongside each batch of samples to check for contamination from the procedure or equipment. Any detected PFAS quantity in the matrix blanks was subtracted from the sample results. To assess the accuracy of the method, spiked matrices were used. A bio‐organic egg homogenate was spiked with a reference solution containing all 17 PFASs. Recoveries from these spiked matrices were within the acceptable range (85%–115%), demonstrating good method precision. Although PFOS showed a slightly higher recovery (121%) in one batch, this value remains within commonly accepted limits and is not expected to bias reported concentrations significantly. Method detection limits (MDLs) were determined by calculating the standard deviation (SD) between eight replicates of a low spike matrix sample that underwent the entire extraction procedure. The MDLs and method reporting limits (MRLs) were established as three times SD and five times MDL, respectively. Each MDL and MRL are corrected for the sample weight.

A second‐source calibration solution, from an interlaboratory study (NCP‐AMP13‐2), was also used to verify calibration accuracy. The results obtained with this calibration solution were compared to the expected concentrations, and these were 101.4% ± 6.4% (mean ± standard deviation [SD]) within the range of 80%–120%. In addition, a random sample from each series was extracted in duplicate to verify the method's precision. The results of the duplicate analyses showed good precision, with relative percent differences (RPDs) of 3.6% ± 2.7%, all less than 20% for samples above the MRL, demonstrating excellent method precision. The raw data and calculations were thoroughly checked to ensure adherence to the method's quality criteria. All values were corrected considering contamination blanks and analysis precision.

### 2.4 Lipid and Moisture Content Analysis

The egg samples were homogenized to ensure an even distribution of components. They were stored frozen at −20°C until thawed for subsampling and analysis. Precisely weighed aliquots were mixed with sodium sulfate to facilitate moisture absorption and phase separation. Lipids were extracted using ultrasonication, a technique that applies high‐frequency sound waves to disrupt cell membranes and release lipids. The extracts were then purified through gel permeation chromatography (GPC) to separate target compounds from residual lipids and biogenic substances. Final purification was performed using solid‐phase extraction (SPE), which removed any remaining lipids that could interfere with the analyses. Lipid and moisture percentages were determined by oven drying, a standard method to remove all water and obtain an accurate measure of the dry lipid content. All procedures followed the methods described by Champoux et al. (2015, 2017). Although PFAS are not considered lipophilic (Buck et al. 2011), lipid normalization was applied to account for empirical correlations observed between PFAS concentrations and lipid content (Table S1). In addition, δ^15^N‐based correction was used to account for trophic‐level effects prior to statistical analyses (see Section 2.6.4).

### 2.5 Stable Isotope Analysis of Nitrogen (δ^15^N) and Carbon (δ^13^C)

Stable isotope ratios of nitrogen (δ^15^N) and carbon (δ^13^C) were determined in aliquots of homogenized northern gannet eggs to assess the trophic position and foraging area of adult females, respectively. The isotopic composition of organic carbon and nitrogen in eggs collected between 1969 and 2004 was measured at the National Hydrology Research Centre, Environment Canada (Saskatoon, Saskatchewan), using flash combustion on a Eurovector elemental analyzer, followed by analysis of the resulting CO_2_ and N_2_ gases on an Isoprime continuous flow isotope‐ratio mass spectrometer (CF‐IRMS) as described in Champoux et al. (2015, 2017). Egg samples from 2009 to 2024 were analyzed at the Ján Veizer Stable Isotope Laboratory (University of Ottawa), formerly known as the G. G. Hatch Stable Isotopes Laboratory. Samples were analyzed using a Varian EL III elemental analyzer coupled to a ConFlo III interface in 2009 (Champoux et al. 2015) or Vario EL Cube elemental analyzer (Elementar, Germany) connected to a Conflo IV interface (Thermo, Germany) in 2012–2024, followed by analysis of the CO_2_ and N_2_ gases on a Delta Plus Advantage CF‐IRMS (ThermoFinnigan; Thermo, Germany). Stable isotope ratios were expressed as deviations from standard reference materials in parts per thousand (‰) and reported in δ notation, using Vienna Pee Dee Belemnite (VPDB) for carbon and atmospheric N_2_ for nitrogen. Internal standard replicates (Pugel protein or glutamic acid) indicated measurement precision below 0.2‰. Measurement precision remained consistent, with typical accuracy better than 0.2‰. The laboratory maintained rigorous QA/QC protocols, including monitoring of randomly duplicated samples, with relative percent differences (RPDs) between duplicates consistently below 5%. Internal standards such as nicotinamide C‐51, ammonium sulfate + sucrose C‐52, caffeine C‐54, and glutamic acid C‐55 were used to ensure isotopic compositions spanned the natural range, and results were calibrated against international standards (IAEA‐N1, IAEA‐N2 for nitrogen, IAEA‐CH‐6, NBS‐22 for carbon).

### 2.6 Statistical Analysis

#### 2.6.1 Handling of Censored Values

Statistical analyses were performed using R Version 4.5.0. For some compounds, measured values fell below the MDL or MRL and were treated as censored. These values were imputed using regression on order statistics (ROS) with the R packages NADA and NADA2 (Lee 2020; Julian and Helsel 2021). The ROS method relies on the distribution of detected values to impute censored observations (Helsel and Lee 2006). Because PFAS concentrations may fluctuate throughout the year, censored values were imputed separately for each year to avoid overestimation or underestimation due to potential temporal variability in contaminant concentrations. Eggs were therefore grouped by year, and each compound/year combination was treated independently to impute censored values based on the uncensored observations within the same group. When more than 50% of observations for a given compound and year were censored, values were reported as “NA”, as estimation becomes statistically unreliable when the majority of data fall below detection limits (Helsel and Lee 2006). Reporting “NA” in these cases ensures transparency and methodological robustness. Sums of concentrations included both detected and ROS‐imputed values. Total ∑_17_PFASs was calculated, followed by sums based on chemical structure: 13 PFCAs (∑_13_PFCAs) and four PFSAs (∑_4_PFSAs).

#### 2.6.2 Correction of the Suess and Laws Effects

The Suess effect reflects changes in stable isotopes of carbon (δ^13^C) caused by anthropogenic CO_2_ emissions since the industrial revolution (Suess 1955), while the Laws effect accounts for variations in stable carbon isotope fractionation by phytoplankton (Henderson et al. 2024). Suess and Laws effects δ^13^C values were corrected using the R package SuessR (Clark et al. 2021), with 1950 set as the reference year, marking the start of the modern industrial era, and specifying the North Atlantic subpolar region. Setting 1950 as the baseline removes the influence of these global changes, ensuring that variations in carbon isotopes in eggs reflect local biological and environmental factors rather than recent industrial CO_2_ emissions. Specifying the North Atlantic subpolar region further accounts for regional oceanographic characteristics, such as circulation patterns and CO_2_ inputs, improving the accuracy of the results by reducing biases from external isotopic variations. The δ^13^C values corrected for the Suess and Laws effects were included as covariates in statistical models to account for dietary variation but were not used to adjust PFAS concentrations.

#### 2.6.3 Generalized Additive Modeling of PFAS Concentrations and Covariate Effects

For each compound, a series of generalized additive models (GAMs) was fitted to evaluate the effects of covariates: year, moisture content, lipid content, δ^15^N, and δ^13^C. Each covariate was modeled linearly using a smoothing spline or excluded, resulting in 243 candidate models, including a null model. GAMs were fitted using restricted maximum likelihood (REML) and ranked by the small‐sample‐corrected Akaike information criterion (AICc). Models with a ΔAICc below two were considered equally plausible (Table S3). When more than one model had a ΔAICc below two, model averaging was performed on models representing up to 95% of the cumulative Akaike weight (Burnham and Anderson 2002). The relative contribution of each covariate was then extracted to assess their importance in explaining PFAS concentrations (Figure 1; Burnham and Anderson 2002; Symonds and Moussalli 2011).

**FIGURE 1:**
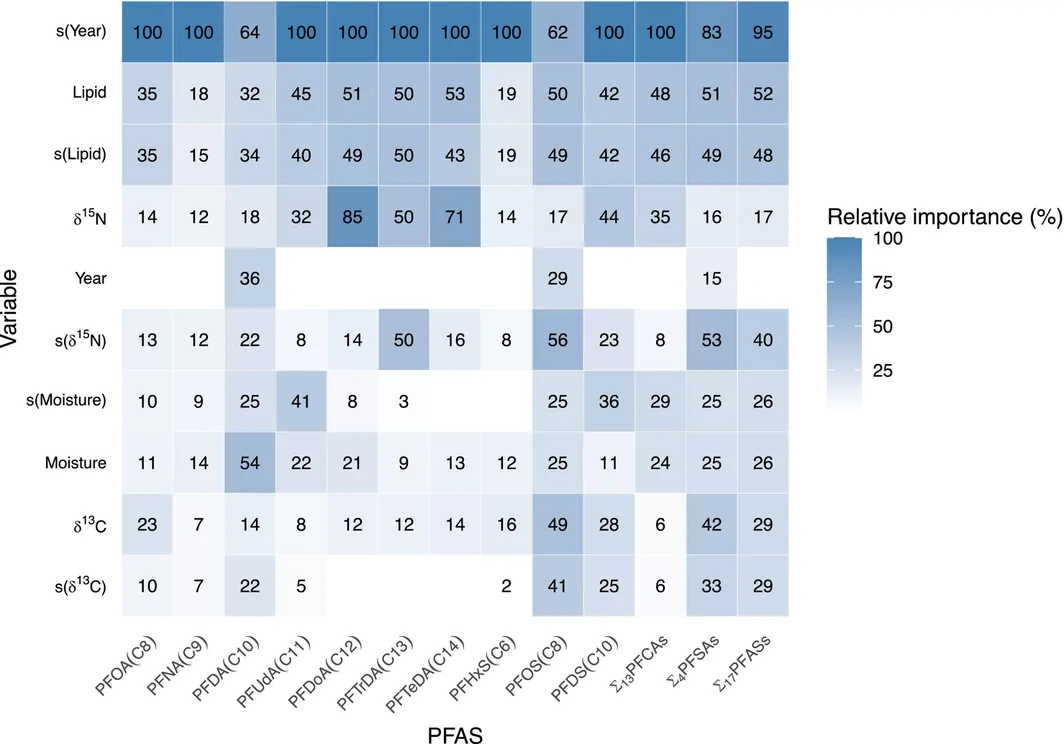
Relative importance (%) of variables in explaining PFAS concentration variability in northern gannet ( *Morus bassanus* ) eggs from Bonaventure Island. Values are based on a subset of models representing up to 95% of the cumulative Akaike weight from generalized additive models (GAMs). Variables are ranked from top to bottom according to their average importance explaining overall PFAS concentrations. Dark blue indicates high relative importance, while lighter shades reflect lower importance. Variables preceded by s() represent nonlinear effects modeled using smoothing functions (splines), while variables without s() indicate linear relationships.

#### 2.6.4 Correction of PFAS Concentrations

Although PFAS are not lipophilic like legacy organic contaminants (e.g., DDT), their amphiphilic properties can lead to partial affinities with lipid fractions, which justifies lipid normalization in the present study. Lipid content may therefore influence PFAS concentrations in eggs, as indicated by the significant relationships observed between lipid content and individual PFAS concentrations (Table S1; Ohoro et al. 2024). To account for potential variations in lipid content and trophic level over time, two datasets of corrected PFAS concentrations were analyzed: one adjusted for lipids only and the other for both lipids and δ^15^N (details are provided in the Supporting Information). Stable nitrogen isotope ratios (δ^15^N) indicate the birds' trophic level. Individuals feeding higher in the food chain accumulate greater amounts of PFAS, as these substances become progressively concentrated at each level through biomagnification (Castillo‐Guerrero et al. 2025). The correlations between these variables and PFAS concentrations justified the applied corrections, which can reduce the noise in the analysis of temporal trends (Figure 1; Table S1).

PFAS concentrations were corrected for lipid content using a linear normalization approach based on their correlation (Figures 1 and S1; Table S1). The formula used is as follows: $C_{\text{corrected}}=C_{\text{measured}}+b\times \left(L_{\max}-L_{\text{sample}}\right)$, where *C*_corrected_ is the corrected concentration of the contaminant (ng/g wet weight [ww]), *C*_measured_ is the raw concentration value obtained directly from the measurement process (ng/g ww), *b* is the slope of the linear relationship between contaminant concentration and lipid content, *L*_max_ is the maximum lipid content (%), and *L*_sample_ is the lipid content of the sample (%).

Concentrations were also adjusted to account for potential shifts in trophic level over time and their effect on PFAS biomagnification (Figure 1). This approach used trophic magnification factors (TMFs) from Miranda et al. (2021), who compiled TMFs from multiple studies for freshwater and marine systems. For marine ecosystems, average TMF values were prioritized, while freshwater values were used when marine data were unavailable. Trophic magnification slopes (TMSs) were then calculated to quantify the increase in concentration per unit change in δ^15^N, enabling recalculation of concentrations standardized for trophic level (Table S2). The formula used is as follows: *C*_corrected_ = $10^{\left(C_{\text{measured}}+\mathrm{TM}S_{\delta^{15}N}\times \left(\delta^{15}N_{\text{mean}}-\delta^{15}N_{\text{sample}}\right)\right)}$, where *C*_*corrected*_ is the corrected concentration of the contaminant (ng/g ww), *C*_*measured*_ is the measured concentration of the contaminant (ng/g ww), ${\mathrm{TMS}}_{\delta^{15}N}$ is the Trophic Magnification Slope (TMS) based on stable nitrogen isotope ratios, specifically, *δ* ^*15*^ *N*_*sample*_ is the measured value of δ^15^N of the sample (‰), and, *δ* ^*15*^ *N*_*mean*_ is the average value of δ^15^N for all years (‰). These corrections were applied on raw (i.e., nontransformed) concentrations, and all concentrations are expressed on a wet‐weight basis.

#### 2.6.5 Temporal Trends, Relative Contributions, and Ecological Half‐Lives

To model temporal trends, another set of GAMs was individually applied to each compound or sum, incorporating spline smoothing. To identify periods of significant changes in concentrations, the derivatives of these GAMs were then calculated, with 95% confidence intervals (±95% CI). A change was considered statistically significant when CIs did not overlap with zero (Figure S2). To determine the peak concentration, a point where the derivative was equal to zero, along with its 95% CI, was added (details are provided in the Supporting Information).

The relative contribution (%) of each compound to the sum of the 17 analyzed PFASs was calculated to examine their relative variations over time. To assess whether the relative contribution of each PFAS compound changed significantly before and after the year of peak concentrations (as previously mentioned), a Shapiro–Wilk test was performed on both groups to evaluate normality. If both groups followed a normal distribution, a variance test (Fisher test) was used to determine whether to apply a standard *t*‐test (if the variances were equal) or Welch's *t*‐test (if the variances were different). If at least one of the two groups did not follow a normal distribution, a nonparametric Wilcoxon test was used. A *p* value < 0.05 was used to indicate a significant difference (Table S5).

For each compound, the ecological half‐life was estimated by adjusting a logarithmic regression of lipid‐corrected concentrations and double‐corrected (lipids and δ^15^N) concentrations over time, from the peak year to 2024. The ecological half‐life of a contaminant corresponds to the time required for its concentration to decrease by 50% from peak level. This measure integrates all biotic and abiotic factors, as well as both input and removal processes that influence observed concentrations (Hebert et al. 1999). The half‐life of PFAS was calculated using the following formula (adapted from Tardiff and Katzman 2007): $T_{1⁄2}=\frac{\ln\left(2\right)}{\left|\text{slope}\right|}$

## 3 Results

### 3.1 Variables Influencing PFAS Accumulation

Prior to analyzing temporal trends, correlations were assessed between PFAS concentrations (Table 1) and key variables potentially influencing their accumulation in eggs, namely, the year of collection, stable isotopes of carbon (δ^13^C) and nitrogen (δ^15^N), lipid content, and moisture content. This evaluation allowed a clearer interpretation of temporal variations related to industrial activity and regulatory measures (Figure 1).

The relative importance of variables showed that year was the predominant factor explaining the variability in PFAS compound concentrations reaching 100% for nearly all individual compounds as well as for PFAS sums. Lipid content was important as its contribution exceeded 40% for PFUdA, PFDoA, PFTrDA, PFTeDA, PFOS, and PFDS, as well as for PFAS sums (∑_13_PFCAs, ∑_4_PFSAs, ∑_17_PFASs). Similarly, δ^15^N values showed notable relative importance, particularly for PFDoA and PFTeDA. Lastly, moisture content played a moderate role, especially for PFDA and PFUdA. The importance of variables related to δ^13^C values was generally lower but reached up to 49% for PFOS (Figure 1).

### 3.2 Evolution of the Relative Contributions of PFAS Over Time

Among the 17 PFASs analyzed, PFOS remained the dominant compound, accounting for 60%–70% in 1969, then slightly decreasing to about 55% in 2024. PFUdA was the second most dominant compound, increasing from about 9% to 11% in 1969 to 13% in 2024. PFTrDA followed, rising from 6% to 7% to nearly 8%, while PFNA increased from 5% to over 7% during the same period. Other compounds such as PFDA, PFDS, PFDoA, and PFTeDA remained below 5% throughout the entire record, while PFHxS and PFOA remained marginal, contributing less than 1% across all years (Figure 2). PFOS, PFNA, PFUdA, PFDoA, PFTeDA, and PFHxS exhibited significant decreases in relative contribution after their inflection points, whereas PFOA, PFDA, PFTrDA, and PFDS did not show any notable variation (*p* < 0.05; Table S5).

**FIGURE 2:**
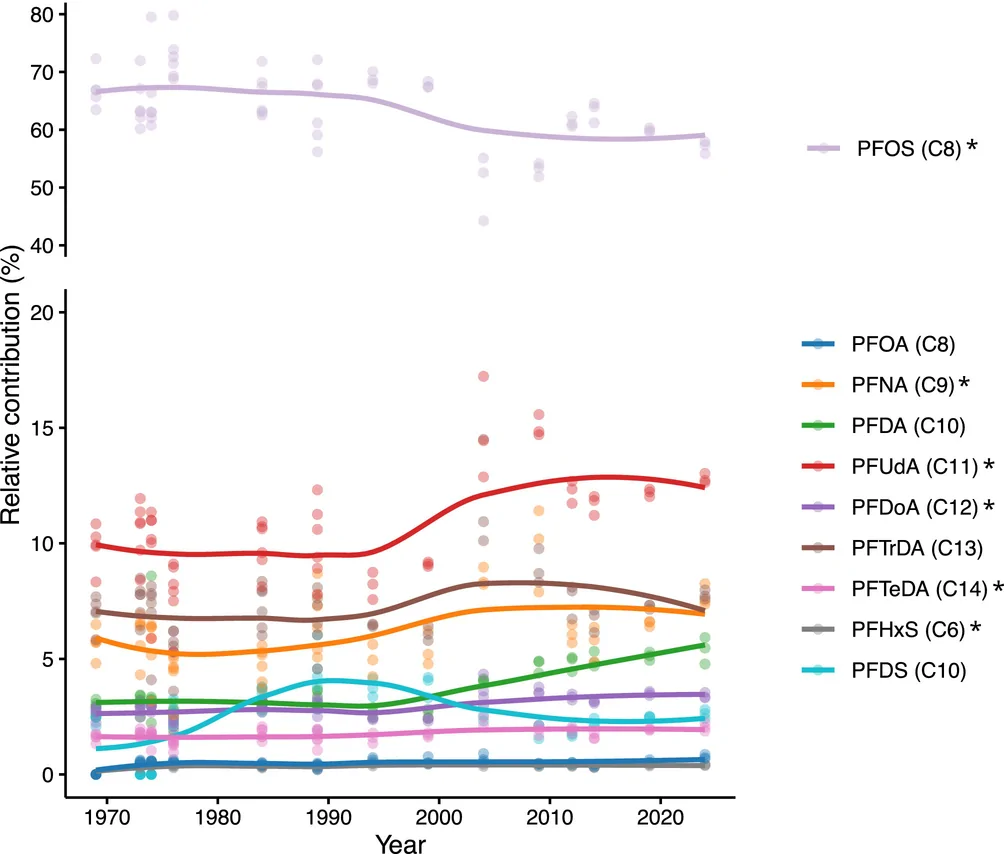
Relative contribution of each PFAS compound to the sum of the 17 analyzed PFASs (∑_17_PFASs) over time in northern gannet ( *Morus bassanus* ) eggs from Bonaventure Island. Compounds below the detection limit were excluded from the figure but included in the calculation of total PFAS. An asterisk (*) indicates a significant change in relative contribution before and after the year of peak concentration of each compound (*p* < 0.05; Table S5).

### 3.3 Temporal Trends in PFAS Concentrations

PFSA compounds generally exhibited a pattern of increase, peak, and gradual decline, consistent across both models (lipid‐corrected and lipid‐δ^15^N‐corrected models). PFHxS, PFDS, and PFOS increased until the mid‐1990s and then declined from the late 1990s or early 2000s until the 2020s. ∑_4_PFSAs followed a similar trend, peaking around the mid‐1990s. PFOS concentrations exceeded the PFOS‐specific LOAEL from approximately 1980–2010, while the combined PFOS + PFHxS concentrations surpassed the LOAEL for the PFOS:PFHxS mixture during the same period (Figures 3 and S3; Table S4).

**FIGURE 3:**
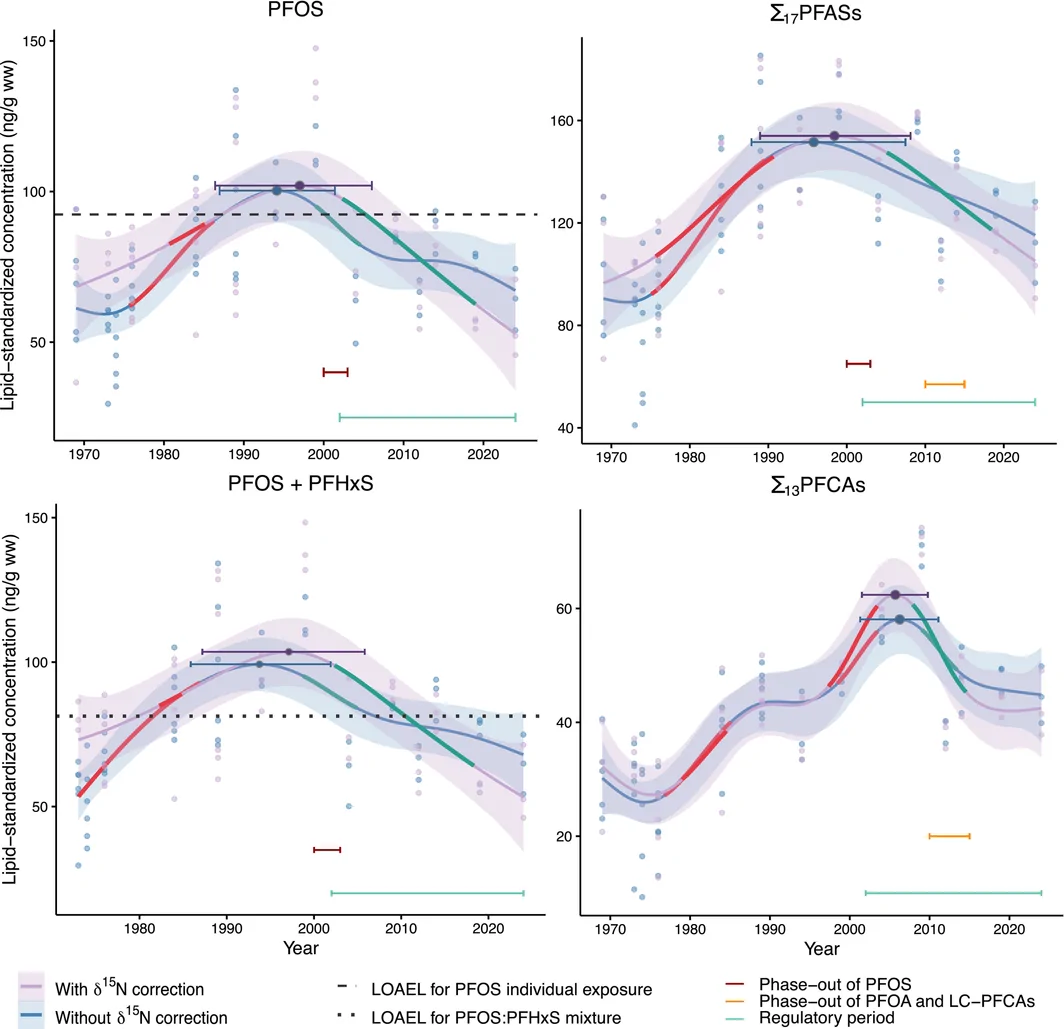
Temporal trends in PFAS concentrations (ng/g wet weight) in northern gannet ( *Morus bassanus* ) eggs from Bonaventure Island: PFOS, PFOS + PFHxS, ∑_17_PFASs and ∑_13_PFCAs. Two trend curves are shown: in purple, concentrations corrected for both lipid content and stable nitrogen isotope ratios (δ^15^N); in blue, concentrations corrected for lipid content only. Likewise, inflection points are displayed with (purple) and without (blue) δ^15^N correction. Colored segments highlight significant increases (red) and decreases (green). Lowest observable adverse effect level (LOAEL) thresholds are indicated for PFOS (92.4 ng/g wet weight) and for a PFOS:PFHxS mixture (81.3 ng/g wet weight; Dennis et al. 2022). Colored bars represent periods of voluntary production phase‐out and regulation in North America: PFOS phase‐out (2000–2003; dark red), PFOA and long‐chain PFCAs (LC‐PFCAs) phase‐out (2010–2015; orange), and regulatory period (2002–2024; green).

PFCA compounds displayed more heterogeneous temporal patterns, often with two phases of increase: the 1970s–1980s and the late 1990s–early 2000s (Figure 3). Most peaked between 2004 and 2009, except PFOA (C8), which peaked around 1999–2000, and PFDA (C10), which showed a nearly linear increase without a clear peak in the lipid‐corrected model (Figure S3). The sum ∑_17_PFASs peaked between 1996 and 1999, largely driven by PFOS (Figures 2 and 3; Table S4). Estimated ecological half‐lives in northern gannet eggs provide insight into PFAS persistence in the environment. In the lipid‐corrected models, half‐lives ranged from 28 years (PFDS) to 85 years (PFOA) and from 17 to 51 years after lipid and δ^15^N correction. Half‐lives tended to be higher in the lipid‐corrected model than in the model corrected for both lipids and δ^15^N, indicating that accounting for both lipid content and trophic position generally reduces the estimated persistence of these compounds (Table 2).

**TABLE 2:** Estimated ecological half‐lives of PFAS^a^ in northern gannet ( *Morus bassanus* ) eggs from Bonaventure Island, from peak concentrations to 2024.

Compound	Lipid correction	Lipid and δ^15^N correction
PFHxS (C6)	41	38
PFOS (C8)	54	21
PFDS (C10)	28	17
∑_4_PFSAs	52	27
PFOA (C8)	85	51
PFNA (C9)	36	24
PFDA (C10)	ND	32
PFUdA (C11)	34	25
PFDoA (C12)	47	34
PFTrDA (C13)	35	27
PFTeDA (C14)	52	35
∑_13_PFCAs	41	31
∑_17_PFASs	60	36

Abbreviation: ND, not detected.

^a^ PFBS, PFBA, PFPeA, PFHxA, PFHpA, PFHxDA, and PFODA did not yield data or sufficient data above detection limits.

## 4 Discussion

### 4.1 Variables Influencing PFAS Accumulation

Among detected PFASs, PFOS (C8; PFSA) was by far the compound with the highest relative contribution in northern gannet eggs, reflecting its historically widespread use, environmental persistence, and strong affinity for proteins and to a lower extent for lipids (see Section 4.2). In addition to PFOS, several long‐chain PFCAs, namely, PFNA (C9), PFUdA (C11), and PFTrDA (C13), also exhibited substantial relative contributions, ranking after PFOS. Although PFAS are known to preferentially bind to proteins, they also exhibit affinity for lipids. This lipid affinity increases with perfluorinated chain length, thereby promoting membrane association, tissue retention, and bioaccumulation (Miranda et al. 2021; Ohoro et al. 2024). In contrast, short‐chain PFASs (e.g., PFBA, PFPeA, PFHxA, PFHpA, PFBS) were generally below detection limits, which aligns with their reduced hydrophobicity, limited lipid affinity, and low propensity for biomagnification. Similarly, some long‐chain PFASs (e.g., PFHxDA, PFODA) showed minimal accumulation, likely due to their large molecular size hindering transfer across biological membranes, which explains their rare detection in eggs (Miranda et al. 2021).

Sampling year emerged as the main variable explaining PFAS concentrations across compounds (Figure 1), reflecting temporal variations in production, regulation, and environmental inputs (Abunada et al. 2020). Lipid content was also a strong predictor for several long‐chain PFASs, including PFDoA, PFTrDA, PFTeDA (PFCAs), and PFOS (PFSA), as shown by the high importance of both “Lipid” and “s (Lipid)” terms. This agrees with known physicochemical behavior, as longer perfluorinated chains (≥C8 for PFCA; ≥C6 for PFSA) increase affinity for lipid membranes, facilitating retention and accumulation in tissues (Ohoro et al. 2024). PFSA compounds, including PFOS, additionally exhibit strong binding affinity for plasma proteins due to their sulfonate group (–SO_3_^−^), which binds to positively charged protein sites such as albumin (Forsthuber et al. 2020; Ohoro et al. 2024). This combined protein‐ and lipid‐binding behavior likely contributes to their high persistence in apex predators.

Trophic position, estimated via δ^15^N values, also strongly influenced concentrations of the same long‐chain PFASs. Here, δ^15^N and s(δ^15^N) were as important, or even more important than, lipid content, reflecting the effect of biomagnification along food webs. Higher trophic levels consistently showed elevated PFAS concentrations, a pattern widely documented in marine mammals, seabirds, and fish (Alfaro Garcia et al. 2022; Ren et al. 2022; Khalid et al. 2024). The relationship is also consistent with reports of TMFs (TMFs > 1) for compounds such as PFOS and PFNA in marine food webs (Miranda et al. 2021), even though TMFs were not directly measured here. Annual shifts in food web structure (diet, prey availability, species composition) may therefore contribute to interannual variability observed in the dataset. Another study demonstrated similar trophic‐level influences: Kesic et al. (2023) incorporated stable carbon (δ^13^C) and nitrogen (δ^15^N) isotopes to account for dietary effects and refine the interpretation of temporal trends of PFAS in seabird eggs. This approach showed that the marked declines in PFOS between 1973 and 2019 were not solely driven by trophic shifts, confirming a genuine reduction in exposure linked to regulatory restrictions. It emphasizes the importance of including factors such as lipids and trophic variables (e.g., δ^15^N) into PFAS analyses to improve both robustness and interpretability. The influence of δ^13^C was also important in the present study, but to a lower degree and only for PFOS. Along with δ^15^N, this suggests a diet shift of gannets throughout the duration of this study.

### 4.2 Temporal Trends in PFAS Concentrations and Relative Contributions

Between 1969 and the late 2000s, ∑_17_PFASs concentrations in northern gannet eggs exhibited marked fluctuations, peaking in the 1990s before gradually declining, with compound‐specific variations. These patterns reflect both historical widespread industrial uses followed by the implementation of regulations targeting different PFAS compounds. PFOS, the dominant PFAS compound, largely drove the 1990s peak. Its concentrations increased sharply between 1969 and the mid‐1990s, driven by widespread industrial applications, especially in firefighting foams, textile treatments, and various manufacturing processes, in the absence of regulatory constraints (Abunada et al. 2020; Brennan et al. 2021). This extensive use led to a significant increase in environmental emissions and accumulation in aquatic ecosystems, notably the Great Lakes and St. Lawrence River basin (Ren et al. 2022; Khalid et al. 2024; Xia et al. 2024; Teymoorian et al. 2025).

A major turning point occurred in 2000 when the company 3M, the leading PFOS manufacturer, announced a gradual phase‐out in collaboration with the US Environmental Protection Agency (U.S. Environmental Protection Agency (EPA) 2000, 2002). This withdrawal coincided with a rapid decline in PFOS and ∑_4_PFSAs concentrations, as well as a reduced PFOS contribution to the overall ∑_17_PFASs profile (Figures 2 and 3 and S3; Table S4). By 2006, risk management actions for PFOS were under development in Canada, while regulatory restrictions had already been implemented in certain international jurisdictions, including the European Union (Government of Canada 2006; European Parliament and Council of the European Union 2006; United Nations Environment Programme (UNEP) 2009; Table S6). In Canada, federal regulatory prohibition measures for PFOS, its salts, and precursors were established in 2008 and subsequently consolidated within the federal regulatory framework (Government of Canada 2025a; Table S6). In Europe, Directive 2006/122/EC restricted the marketing and use of products containing PFOS, with specific exemptions allowed until 2015 (European Parliament and Council of the European Union 2006). Internationally, PFOS was listed under Annex B of the Stockholm Convention in 2009, which requires signatory countries to restrict its production and use, while allowing certain limited exemptions for specific applications, such as some fire‐fighting foams and industrial uses, to target its restriction and progressive elimination (United Nations Environment Programme (UNEP) 2009). Similar trends in biological tissues have been reported elsewhere. Along the Canadian Pacific coast, Kesic et al. (2023) documented declining PFOS concentrations in eggs of several seabird species since the early 2000s, consistent with the timing of regulatory measures and the phase‐out by 3M and the US EPA. In Europe, Pereira et al. (2021) observed PFOS and ∑PFSAs peaking in northern gannet eggs in the UK between 1994 and 1998, followed by a decline linked to regulatory restrictions. Similarly, Haque et al. (2023) reported a PFOS peak in Swedish white‐tailed sea eagle ( *Haliaeetus albicilla* ) eggs, with subsequent decreases after global production halted. PFDS and PFHxS also showed declines in the present study, despite not being initially regulated, potentially due to co‐production with PFOS or their presence as by‐products (Benskin et al. 2010), suggesting indirect benefits of PFOS restrictions. PFHxS and its related compounds have been recently listed under Annex A of the Stockholm Convention in 2022 (United Nations Environment Programme (UNEP) 2022). The listing reflects the recognition of PFHxS as a persistent, bioaccumulative, and potentially toxic substance, requiring strict global measures for its progressive elimination.

PFCA concentrations, particularly C9–C14, increased significantly in eggs between approximately 2005 and 2010, reflecting a gradual shift from PFSA to PFCA use in the production of fluoropolymers, which are used in products such as nonstick coatings, waterproof textiles, and certain food packaging (Abunada et al. 2020). Concentrations stabilized during the 2010s before declining, likely in response to regulatory measures. Indeed, the US PFOA Stewardship Program (2006) aimed to reduce PFOA emissions by 95% by 2010 and eliminate them by 2015 (U.S. Environmental Protection Agency (EPA) 2025), while Canada implemented environmental performance agreements in 2010 to restrict PFOA and long‐chain PFCAs, preceding formal regulations in 2016 (Government of Canada 2018, 2025b, 2025c; Table S6). Similar trends have been observed in Canada, where Kesic et al. (2023) reported plateauing or slight declines in long‐chain PFCAs after 2010, suggesting that national regulatory measures and global phase‐outs are effective in reducing environmental PFCA concentrations. In Europe, regulatory initiatives such as the EU restrictions and the Stockholm Convention have contributed to similar decreases (European Parliament and Council of the European Union 2006; United Nations Environment Programme (UNEP) 2019). Consistently, Pereira et al. (2021) reported a continuous rise in ∑PFCAs in UK gannet eggs until 2014, while Haque et al. (2023) observed stabilization in Swedish white‐tailed sea eagle eggs after the late 2000s.

### 4.3 Ecological Half‐Lives of PFAS in the Northern Gannet Eggs

PFOS exhibited one of the longest ecological half‐lives, estimated at 54 years after lipid correction and reduced to 21 years when corrected for both lipids and δ^15^N. For instance, Kesic et al. (2023) reported PFOS half‐lives in other seabird species, such as Leach's storm‐petrel (*Hydrobates leucorhous*; 2.6 years) and rhinoceros auklet ( *Cerorhinca monocerata* ; 7.8 years), while Miller et al. (2015) found longer half‐lives in rhinoceros auklet on the southern and northern Pacific coasts (12.7 and 21 years, respectively). In general, species occupying higher trophic levels exhibit longer half‐lives because they accumulate more PFAS due to their higher position in the food chain, which is consistent with current observations. However, these estimates should be interpreted cautiously, as conditions for true decline, such as complete cessation of use and emissions, are not fully met. In reality, several compounds continue to be used and released in the environment. Thus, the half‐lives calculated here do not reflect only intrinsic degradation processes but rather an apparent trend influenced by environmental persistence, bioaccumulation, and changing exposure over time.

Other PFASs, including PFHxS, PFDS, and ∑_4_PFSAs, exhibited long ecological half‐lives, highlighting the strong persistence of PFSA even after lipid and δ^15^N correction. Long half‐lives were also observed for PFCA, particularly in models corrected only for lipids. PFOA initially reached 85 years and remained the longest‐lived PFCA after correction for both lipids and δ^15^N, with an estimated half‐life of 51 years. The persistence of long‐chain PFCAs (≥C8) is primarily due to their chemical stability and bioaccumulation potential, which facilitate accumulation in biological matrices and resistance to biodegradation (Abunada et al. 2020; Ohoro et al. 2024). At present, no studies report ecological half‐lives for PFAS other than PFOS, so comparative data are still limited.

### 4.4 Toxicity

From the 1980s to 2010, concentrations of PFOS and PFOS + PFHxS in gannet eggs exceeded LOAEL thresholds established by Dennis et al. (2022). These thresholds (92.4 ng/g ww for PFOS alone and 81.3 ng/g ww for a PFOS:PFHxS mixture) were based on dietary exposure studies in northern bobwhite quail ( *Colinus virginianus* ). They are associated with severe reproductive effects, including reduced hatching success, embryonic abnormalities, and compromised egg quality. The lasting exceedance indicates that eggs were likely exposed to a high risk of reproductive disruption during this period.

However, applying these thresholds to wild seabirds should be done with caution. First, dietary studies in controlled conditions may not fully reflect environmental exposures, where multiple contaminants occur simultaneously. Second, species‐specific sensitivity matters: some birds are inherently more tolerant, while others are highly sensitive. For example, toxicological studies with AHR agonists (e.g., dioxin‐like compounds) show that birds with highly sensitive AHR1 subtypes, such as domestic chickens (*Gallus gallus domesticus*), are commonly used as model species (Head and Kennedy 2007). In contrast, species expressing less sensitive AHR1 subtypes exhibit reduced sensitivity to dioxin‐like compounds (Bianchini and Morrissey 2020). Consequently, reproductive risks inferred from highly sensitive models such as quail may overestimate sensitivity in seabirds with different physiological and ecological traits, although uncertainties remain.

Total ∑_17_PFASs concentrations also surpassed these thresholds, despite the absence of established toxicity thresholds for several of the compounds included. Given that the PFOS:PFHxS mixture induces stronger effects than PFOS alone, it is reasonable to assume that the presence of 17 PFASs could result in cumulative or synergistic toxicity. The co‐occurrence of multiple PFAS and other families of molecules such as other halogenated contaminants (e.g., polybrominated diphenyl ethers) at elevated concentrations likely exacerbates harmful effects on seabird reproduction.

Beyond reproductive impacts, PFAS, particularly PFOS, affect seabird physiology more broadly. Documented effects include endocrine and immune disruption, impaired liver metabolism, reduced fertility, and altered egg composition and structure (Pereira et al. 2021; Groffen et al. 2024). Chronic exposure may also lead to long‐term effects such as telomere shortening (a marker of premature cellular aging; Sebastiano et al. 2023), neurodevelopmental disorders (Robuck et al. 2021), as well as immunosuppression, which increases vulnerability to infections (Miller et al. 2015; Sebastiano et al. 2023).

## 5 Conclusions

This study, spanning 55 years of research, highlights the evolution of concentrations of 17 PFASs in northern gannet eggs from the Gulf of St. Lawrence. The results show that temporal trends reflect both past and current industrial uses, as well as the progressive implementation of regulatory measures at national and international levels. PFOS, the predominant compound among the 17 PFASs analyzed, exhibited a sharp increase until the mid‐1990s, followed by a decline associated with efforts to reduce and restrict its use. Other PFSAs showed similar dynamics, while long‐chain PFCAs displayed a later increase, reflecting a shift in uses before slowing down in recent years. Notably, PFOS concentrations exceeded the PFOS‐specific LOAEL from approximately 1980–2010, and combined PFOS + PFHxS concentrations surpassed the LOAEL for the PFOS:PFHxS mixture during the same period, indicating that eggs were likely exposed to a high risk of reproductive disruption.

Overall, the observed trends confirm the gradual effectiveness of management actions, while underlining the high environmental persistence of PFAS compounds. The estimation of ecological half‐lives indicates that the decline in concentrations is slow, emphasizing the importance of maintaining scientific and regulatory vigilance. The high ecological half‐lives estimated here show the persistence of these molecules and suggest that they will be detected in the next decades, like what has been found for legacy contaminants such as DDT and polychlorinated biphenyls (PCBs; Chapdelaine et al. 1987; Elliott et al. 1988; Champoux et al. 2015, 2017). Continued long‐term research, therefore, remains essential to document the evolution of these substances, to evaluate the effectiveness of measures already implemented, and to support future environmental management decisions.

## Funding

This work was supported by the Environment and Climate Change Canada.

## Conflicts of Interest

The authors declare no conflicts of interest.

## Supporting information


**Table S1:** Linear regression parameters describing the relationship between PFAS concentrations and lipid content across samples.
**Table S2:** TMF_TL_ values for individual PFAS compounds and PFAS sums along with the associated $\mathrm{TM}S_{\delta^{15}N}$ values.
**Table S3:** Comparison of top (∆AICc < 2) generalized additive models (GAMs) for each compound, including corresponding AICc, ∆AICc, weight, *R* ^2^, and null model parameters (AICc, weight, inclusion in top models).
**Table S4:** Year of maximum concentrations and associated concentrations, periods of significant increase and decrease (and associated concentration ranges), and ecological half‐lives of PFAS, corrected for lipid content and for both lipid content and stable nitrogen isotope ratios (δ^15^N).
**Table S5:** Statistical comparison of the relative contributions of individual PFAS compounds before and after their peak year.
**Table S6:** PFAS regulations in the studied region and internationally: regulations/limits, responsible organization or country, effective date, targeted PFAS substances, and references.
**Figure S1:** Relationship between PFOS concentrations corrected using Method 1 and Method 2.
**Figure S2:** Temporal derivatives of lipid‐standardized PFOS concentrations and identification of significant trends.
**Figure S3:** Temporal trends in PFAS concentrations in northern gannet eggs from Bonaventure Island: PFOA, PFNA, PFDA, PFUdA, PFDoA, PFTrDA, PFTeDA, PFHxS, ∑_4_PFSAs, and PFDS.

## Data Availability

The raw data and R scripts used to process the data are publicly available on GitHub at: https://github.com/ECCC‐lavoie‐ecotox/NOGA‐PFAS‐1969‐2024.
