# Supplementary material for: Half a Century of Per‐ and Polyfluoroalkyl Substances in Northern Gannet Eggs: Impact of Regulations
Source: J Appl Toxicol. 2026 Mar 27;46(11):4085–98. doi: 10.1002/jat.70175 (PMC13628432; doi:10.1002/jat.70175)
Supplement: Supplementary file 1 [file JAT-46-4085-s001.docx]

**Supporting information for**

Half a century of per- and polyfluoroalkyl substances in northern gannet eggs: Impact of regulations

Anaïs Fournier^a,b^, Anaïs Kerric^b^, Raphaël A. Lavoie^b,*^

^a^ Department of Chemistry, University of Bordeaux, 351 cours de la Libération, 33405 Talence, France

^b^ Science and Technology Branch, Environment and Climate Change Canada, 801-1550 Av. d’Estimauville, Québec, Québec, Canada, G1J 0C3

* Corresponding author: [raphael.lavoie@ec.gc.ca](mailto:raphael.lavoie@ec.gc.ca)

This supporting file includes: 26 pages, 6 tables and 3 figures.

|  | Description | Page |
| --- | --- | --- |
| Table S1 | Linear regression parameters describing the relationship between PFAS concentrations (ng/g wet weight (ww)) and lipid content (%) across samples. For each PFAS compound, the intercept, slope, and associated p-value are reported. Non-detects were imputed prior to the analysis to ensure sufficient data availability. | S4 |
| Figure S1 | Relationship between PFOS concentrations corrected using Method 1 (ng/g lipid) and Method 2 (ng/g ww). This plot illustrates the strong linear relationship between the two correction approaches applied to PFOS concentrations, with a coefficient of determination R^2^ = 0.849, indicating high consistency between methods. | S6 |
| Table S2 | TMF_TL_ values (from Miranda *et al.*, 2021) for individual PFAS compounds and PFAS sums along with the associated $TMS_{\delta^{15}N}$ values. | S8 |
| Table S3 | Comparison of top (∆AICc < 2) Generalized Additive Models (GAMs) for each compound, including corresponding AICc, ∆AICc, Weight, R^2^, and null model parameters (AICc, weight, inclusion in top models). | S9 |
| Figure S2 | Temporal derivatives (f’(x)) of lipid-standardized PFOS (the compound with the highest relative contribution) concentrations and identification of significant trends. This figure shows the rate of change in PFOS concentrations over time, after correction for lipid content. The blue line represents the estimated temporal trend, surrounded by its 95% confidence interval. Sections where the confidence interval lies entirely below zero are shown in green, indicating a significant decreasing trend. Conversely, sections where the confidence interval lies entirely above zero are shown in red, indicating a significant increasing trend. | S12 |
| Table S4 | Year of maximum concentrations [year–95% CI - year+95% CI] and associated concentrations [concentration–95% CI - concentration+95% CI], periods of significant increase and decrease (and associated concentration ranges), and ecological half-lives of PFAS, corrected for lipid content and for both lipid content and stable nitrogen isotope ratios (δ^15^N). | S13 |
| Figure S3 | Temporal trends in PFAS concentrations (ng/g ww) in northern gannet (*Morus bassanus*) eggs from Bonaventure Island: PFOA, PFNA, PFDA, PFUdA, PFDoA, PFTrDA, PFTeDA, PFHxS, ∑_4_PFSAs and PFDS. Two trend curves are shown: in purple, concentrations corrected for both lipid content and stable nitrogen isotope ratios (δ^15^N); in blue, concentrations corrected for lipid content only. Likewise, inflection points are displayed with (purple) and without (blue) δ^15^N correction. Colored segments highlight significant increases (red) and decreases (green). Colored bars represent periods of voluntary production phase-out and regulation in North America: PFOS phase-out (2000–2003; dark red), PFOA and long-chain PFCAs (LC-PFCAs) phase-out (2010–2015; orange), and regulatory period (2002–2024; green). | S15 |
| Table S5 | Statistical comparison of the relative contributions of individual PFAS compounds before and after their peak year. For each compound, the table reports the year of maximum concentration, results of Shapiro-Wilk tests for normality before and after the peak (p-values), the Levene’s test for equality of variances, the statistical test used to compare the two periods, and the corresponding p-value. The table also includes the median relative contributions (%) of each compound before and after the maximum year. | S18 |
| Table S6 | PFAS regulations in the studied region and internationally: regulations/limits, responsible organization or country, effective date, targeted PFAS substances, and references. | S19 |

1. **Correction of concentrations based on lipid content**

Measured concentrations (after imputing the non-detected values) were found to be significantly correlated with lipid content (%; p-value < 0.05; Table S1). Furthermore, lipid content and its smoothed effect s(Lipid) were identified as important variables for several compounds, with a contribution exceeding 40% for certain PFASs and for PFAS sums, highlighting the need for lipid-based correction (Figure 1) [1]. To remove this correlation effect, two correction methods were tested: 1- normalization of concentrations to lipid content and 2- adjustment of concentrations to the maximum lipid content observed among the samples.

**Table S1.** Linear regression parameters describing the relationship between PFAS concentrations (ng/g ww) and lipid content (%) across samples. For each PFAS compound, the intercept, slope, and associated p-value are reported. Non-detects were imputed prior to the analysis to ensure sufficient data availability.

| Compound | Intercept | Slope | p-value |  |
| --- | --- | --- | --- | --- |
| PFOA | -0.61 | 0.22 | 1.57e-05 | |
| PFNA | -10.32 | 3.18 | 1.77e-05 | |
| PFDA | -3.87 | 1.47 | 2.79e-04 | |
| PFUdA | -15.84 | 5.15 | 2.87e-06 | |
| PFDoA | -3.86 | 1.32 | 1.41e-06 | |
| PFTrDA | -11.51 | 3.62 | 3.47e-07 | |
| PFTeDA | -2.45 | 0.81 | 6.45e-07 | |
| PFHxS | -0.51 | 0.17 | 4.17e-06 | |
| PFOS | -58.62 | 24.14 | 3.23e-06 | |
| PFDS | -3.74 | 1.36 | 1.01e-03 | |
| ∑_17_PFASs | -111.81 | 41.48 | 1.90e-07 | |
| ∑_13_PFCAs | -48.56 | 15.80 | 1.52e-06 | |
| ∑_4_PFSAs | -63.25 | 25.68 | 3.15e-06 | |

**Method 1: Correction by lipid normalization**

The most commonly used method to adjust concentration based on lipid content is to divide the measured concentration by the lipid content [2] [3]:

$$C_{corrected}=\frac{C_{measured}}{L}\times100$$

Where:

- *C_corrected_* is the corrected concentration of the contaminant (ng/g lipid),
- *C_measured_* is the measured concentration of the contaminant (ng/g ww),
- *L* is the lipid content (%).

However, this approach modifies both the scale and the unit of the values, making comparison with uncorrected PFAS concentrations reported in the literature more challenging.

**Method 2: Correction by adjustment to the maximum lipid content**

To overcome these limitations, a second method was used, consisting of adjusting the concentrations along the relationship between concentration and lipid content to a common lipid content value [4]. The maximum lipid content was chosen as the target value, because an adjustment based on the average lipid content would have otherwise resulted in negative values.

The initial relationship between the measured concentration and the lipid content is:

$$C=a+b\times L$$

Where:

- *C* is the concentration of the contaminant (ng/g ww),
- *L* is the Lipid content (%),
- *a* is the intercept of the linear relationship,
- *b* is the slope of the linear relationship.

For a given sample:

$$C_{measured}=a+b\times L_{sample}$$

If this relationship is adjusted to the maximum lipid content (L_max_):

$$C_{max}=a+b\times L_{max}$$

To correct the measured concentration (C_measured_) based on the maximum lipid content, the difference due to lipid content is adjusted as follows:

$$C_{corrected}=C_{measured}+\left( C_{max}-C_{measured} \right)$$

Replacing with the equations gives:

$$C_{corrected}=\left( a+b\times L_{sample} \right)+[\left( a+b\times L_{max} \right)-\left( a+b\times L_{sample} \right)]$$

Which leads to:

$$C_{corrected}= a + b \times L_{sample}+ a + b \times L_{max} - a - b \times L_{sample}$$

Thus, the final formula becomes:

$$C_{corrected}=C_{measured}+b\times\left( L_{max}-L_{sample} \right)$$

This method allows for correcting the lipid effect without modifying the concentration unit, facilitating comparisons with uncorrected concentrations reported in the literature.

Furthermore, the corrected PFOS concentrations (the compound with the highest relative contribution) obtained with this method are correlated with those obtained with the method 1 (R^2^ = 0.849; Figure S1).

**Figure S1.** Relationship between PFOS concentrations corrected using Method 1 (ng/g lipid) and Method 2 (ng/g ww). This plot illustrates the strong linear relationship between the two correction approaches applied to PFOS concentrations, with a coefficient of determination R^2^ = 0.849, indicating high consistency between methods.

2. **Correction of concentrations based on the stable nitrogen isotope ratio (δ^15^N)**

Since trophic level has been found to predict PFAS concentrations via a process called biomagnification [5], and because food web structures may vary from year to year, concentrations were adjusted according to δ^15^N values, a proxy for trophic level. This correction highlights interannual variations of environmental PFAS rather than variations due to trophic position [5]. Both δ^15^N and its smoothed effect s(δ^15^N) displayed notable relative importance in explaining PFAS concentration variability in northern gannet eggs from Bonaventure Island, particularly for PFDoA and PFTeDA (Figure 1), indicating that trophic position strongly influences PFAS accumulation and justifying the use of δ^15^N for correction [1].

The following are important distinctions:

- *TMS_TL_* (Trophic Magnification Slope based on Trophic Level) represents the slope of the relationship between contaminant concentration and trophic level in a regression analysis,
- $TMS_{\delta^{15}N}$ (Trophic Magnification Slope based on δ^15^N) corresponds to the slope of the relationship between δ^15^N and the logarithm of contaminant concentration,
- On the other hand, *TMF_TL_* (Trophic Magnification Factor based on Trophic Level) is the trophic magnification factor calculated as the antilogarithm of the TMS, representing the average biomagnification per TL across the entire food web. Unitless, it is the food-web-scale equivalent of the biomagnification factor (BMF), which is simply the ratio of a contaminant's concentration in a predator to that measured in its prey.
- $TMF_{\delta^{15}N}$(Trophic Magnification Factor based on δ^15^N) is similar, but instead relies on δ^15^N as a proxy for trophic position (δ^15^N values increase with higher trophic levels),
- The key difference between *TMS* and *TMF* is that TMF values quantify the degree of biomagnification as a factor (unitless), while TMS values describe the trend or rate of increase in the logarithm of contaminant concentration with increasing trophic level or δ^15^N (expressed as a regression slope).

The concentrations were corrected based on the δ^15^N isotopic ratio using the TMF_TL_ reported by Miranda *et al.* (2021). Average TMF_TL_ values from marine ecosystems were prioritized. When a value was unavailable for a specific compound, TMF_TL_ values from freshwater ecosystems were used instead. The TMF_TL_ were calculated based on δ^15^N to have the $TMS_{\delta^{15}N}$ based on δ^15^N to adjust PFAS concentrations according to the trophic level of northern gannets, using δ^15^N as a proxy for trophic level instead of the trophic level (TL).

The equation used is: $log\left( contaminant \right)=a+b\times TL$

The TMF is then obtained as: $TMF_{TL}={10}^{b}$

The equation relating TL and δ^15^N is: $TL=TL_{base}+\frac{\delta^{15}N_{consumer}- \delta^{15}N_{base}}{\Delta^{15}N}$

Where:

- *TL_base_* is the trophic level of the primary producer (often 1),
- *δ^15^N_consumer_* is the measured value for the studied organism,
- *δ^15^N_base_* is the reference value for the base of the food web, and,
- *Δ^15^N* is the trophic enrichment factor (TEF) (typically 3.4‰).

By substituting TL in the TMF equation:

$$log(contaminant)=a+b\times\left( TL_{base}+\frac{\delta^{15}N_{consumer}-\delta^{15}N_{base}}{\Delta^{15}N} \right)$$

Which becomes:

$$log(contaminant)=a+b\times TL_{base}+b\times\frac{\delta^{15}N_{consumer}}{\Delta^{15}N}-b\times\frac{\delta^{15}N_{base}}{\Delta^{15}N}$$

Therefore:

$$log\left( contaminant \right)=\left( a+b\times TL_{base}-b\times\frac{\delta^{15}N_{base}}{\Delta^{15}N} \right)+\frac{b}{\Delta^{15}N}\times\delta^{15}N_{consumer}$$

The new slope of interest is: $b^{'}=\frac{b}{\Delta^{15}N}$

To find a relationship between log(contaminant) and δ^15^N_consumer_:

- New intercept: $a^{'}=a+b\times TL_{base}-b\times\frac{\delta^{15}N_{base}}{\Delta^{15}N}$
- New slope: $b^{'}=\frac{b}{\Delta^{15}N}$

Thus, the TMS based on δ^15^N becomes: $TMS_{\delta^{15}N}$= $\frac{b}{\Delta^{15}N}$

${TMF}_{TL}$= ${10}^{b}$ $\to$ b = ${log}_{10}({TMF}_{TL})$

So: $TMS_{\delta^{15}N}$= $\frac{{log}_{10}\left( {TMF}_{TL} \right)}{3.4}$

**Table S2.** TMF_TL_ values (from Miranda *et al.*, 2021) for individual PFAS compounds and PFAS sums along with the associated $TMS_{\delta^{15}N}$values.

| Compound | ${TMF}_{TL}$ | $TMS_{\delta^{15}N}$ |
| --- | --- | --- |
| PFOA | 3.2 | 0.149 |
| PFNA | 2.6 | 0.122 |
| PFDA | 4.8 | 0.200 |
| PFUdA | 2.1 | 0.095 |
| PFDoA | 1.7 | 0.068 |
| PFTrDA | 1.7 | 0.068 |
| PFTeDA | 1.6 | 0.060 |
| PFHxS | 1.1 | 0.012 |
| PFOS | 5.2 | 0.211 |
| PFDS | 4.3 | 0.186 |
| ∑_17_PFASs | 1.9 | 0.082 |
| ∑_13_PFCAs | 1.7 | 0.068 |
| ∑_4_PFSAs | 2.8 | 0.132 |

The TMF_TL_ of the sums corresponds to the average of the TMF_TL_ of the individual PFAS that make up the sums:

- *∑_17_PFASs* include all 17 PFASs compounds,
- *∑_13_PFCAs* include PFBA, PFPeA, PFHxA, PFHpA, PFOA, PFNA, PFDA, PFUdA, PFDoA, PFTrDA, PFTeDA, PFHxDA, PFODA,
- *∑_4_PFSAs* include PFBS, PFHxS, PFOS, PFDS.

Finally, to correct the PFAS concentrations, the formula used is:

C_corrected_ = ${10}^{(C_{measured}+ TMS_{\delta^{15}N} \times(\delta^{15}N_{mean} - \delta^{15}N_{sample}) )}$

Where:

- *C_corrected_* is the corrected concentration of the contaminant (ng/g ww),
- *C_measured_* is the measured concentration of the contaminant (ng/g ww),
- ${TMS}_{\delta^{15}N}$ is the Trophic Magnification Slope (TMS) based on stable nitrogen isotope ratios, specifically δ^15^N,
- *δ^15^N_sample_* is the measured value of δ^15^N of the sample (‰),
- *δ^15^N_mean_* is the average value of δ^15^N for all years (‰).

3. **Comparison of top Generalized Additive Models (GAMs) for each compound**

**Table S3.** Comparison of top (∆AICc < 2) Generalized Additive Models (GAMs) for each compound, including corresponding AICc, ΔAICc, Weight, R^2^, and null model parameters (AICc, weight, inclusion in top models).

| Compound | Models | AICc | ΔAICc | Weight | R^2^ | Null model AICc | Null model weight | Null in top* |
| --- | --- | --- | --- | --- | --- | --- | --- | --- |
| PFOA | s(Year) + Lipid; s(Year) + s(Lipid); s(Year); s(Year) + d13CS | -46.22; -46.21; -45.62; -44.54 | 0; 0.01; 0.6; 1.68 | 0.126; 0.125; 0.093; 0.054 | 0.795; 0.795; 0.781; 0.787 | 3.39 | 2.126249e-12 | FALSE |
| PFNA | s(Year) | 180.22 | 0 | 0.282 | 0.828 | 240.3 | 2.607557e-14 | FALSE |
| PFDA | s(Year) + Moisture; s(Year) + Moisture + Lipid; s(Year) + Moisture + s(Lipid); s(Year); Year + Moisture + s(d13CS); s(Year) + Moisture + s(d13CS); s(Year) + s(Moisture); s(Year) + Lipid; s(Year) + s(Lipid); Year + Moisture + Lipid + s(d15N); Year + Moisture + s(Lipid) + s(d15N); s(Year) + Moisture + s(Lipid) + s(d15N); s(Year) + Moisture + Lipid + s(d15N); s(Year) + s(Moisture) + Lipid; s(Year) + s(Moisture) + s(Lipid); Year + Moisture + Lipid; Year + Moisture + s(Lipid) | 100.4; 100.7; 100.7; 100.79; 101.1; 101.1; 101.17; 101.61; 101.61; 101.71; 101.71; 101.71; 101.71; 101.76; 101.76; 102.06; 102.06 | 0; 0.3; 0.3; 0.39; 0.7; 0.71; 0.77; 1.21; 1.21; 1.31; 1.31; 1.31; 1.31; 1.36; 1.36; 1.66; 1.66 | 0.04; 0.034; 0.034; 0.033; 0.028; 0.028; 0.027; 0.022; 0.022; 0.021; 0.021; 0.021; 0.021; 0.02; 0.02; 0.017; 0.017 | 0.885; 0.892; 0.892; 0.879; 0.872; 0.872; 0.896; 0.884; 0.884; 0.877; 0.877; 0.877; 0.877; 0.901; 0.901; 0.856; 0.856 | 182.35 | 1.045055e-19 | FALSE |
| PFUdA | s(Year) + s(Moisture) + Lipid; s(Year) + s(Moisture) + s(Lipid); s(Year) + Lipid; s(Year) + s(Lipid); s(Year) + Moisture + Lipid; s(Year) + Moisture + s(Lipid); s(Year) + s(Moisture) + Lipid + d15N; s(Year) + s(Moisture) + s(Lipid) + d15N; s(Year) + Lipid + d15N; s(Year) + s(Lipid) + d15N; s(Year) + s(Moisture); s(Year) + Moisture + Lipid + d15N; s(Year) | 180.11; 180.11; 180.31; 180.32; 180.54; 180.54; 180.68; 180.73; 180.96; 181.02; 181.4; 181.83; 181.91 | 0; 0; 0.2; 0.2; 0.43; 0.43; 0.57; 0.62; 0.84; 0.91; 1.29; 1.72; 1.79 | 0.073; 0.073; 0.066; 0.066; 0.059; 0.059; 0.055; 0.054; 0.048; 0.046; 0.038; 0.031; 0.03 | 0.941; 0.941; 0.924; 0.924; 0.93; 0.93; 0.946; 0.946; 0.927; 0.927; 0.936; 0.932; 0.915 | 273.61 | 3.630117e-22 | FALSE |
| PFDoA | s(Year) + Lipid + d15N; s(Year) + s(Lipid) + d15N | 68.53; 68.54 | 0; 0.01 | 0.248; 0.246 | 0.909; 0.909 | 152.56 | 1.402796e-19 | FALSE |
| PFTrDA | s(Year) + Lipid + d15N; s(Year) + Lipid + s(d15N); s(Year) + s(Lipid) + d15N; s(Year) + s(Lipid) + s(d15N) | 112.67; 112.68; 112.68; 112.68 | 0; 0; 0; 0.01 | 0.178; 0.177; 0.177; 0.177 | 0.967; 0.967; 0.967; 0.967 | 238.61 | 7.996969e-29 | FALSE |
| PFTeDA | s(Year) + Lipid + d15N; s(Year) + s(Lipid) + d15N | -0.14; -0.14 | 0; 0.01 | 0.246; 0.245 | 0.95; 0.95 | 110.25 | 2.629411e-25 | FALSE |
| PFHxS | s(Year); s(Year) + Lipid; s(Year) + s(Lipid) | -83.51; -81.81; -81.8 | 0; 1.7; 1.71 | 0.275; 0.118; 0.117 | 0.851; 0.856; 0.856 | -21.15 | 7.939842e-15 | FALSE |
| PFOS | s(Year) + Lipid + s(d15N) + d13CS; s(Year) + s(Lipid) + s(d15N) + d13CS; s(Year) + Lipid + d13CS; s(Year) + s(Lipid) + d13CS; Year + Lipid + s(d15N) + d13CS; Year + s(Lipid) + s(d15N) + d13CS; Year + Lipid + s(d15N) + s(d13CS); Year + s(Lipid) + s(d15N) + s(d13CS); s(Year) + Lipid + s(d13CS); s(Year) + s(Lipid) + s(d13CS); Year + Moisture + Lipid + s(d15N) + d13CS; Year + s(Moisture) + Lipid + s(d15N) + d13CS; Year + s(Moisture) + Lipid + s(d15N) + s(d13CS); Year + Moisture + Lipid + s(d15N) + s(d13CS); s(Year) + Lipid + d15N + d13CS; s(Year) + Lipid + d15N + s(d13CS); s(Year) + s(Lipid) + d15N + d13CS; s(Year) + s(Lipid) + d15N + s(d13CS); Year + s(Moisture) + s(Lipid) + s(d15N) + d13CS; Year + s(Moisture) + s(Lipid) + s(d15N) + s(d13CS); Year + Moisture + s(Lipid) + s(d15N) + d13CS; Year + Moisture + s(Lipid) + s(d15N) + s(d13CS) | 369.22; 369.22; 369.79; 369.79; 369.82; 369.82; 369.82; 369.82; 369.85; 369.89; 370.93; 370.93; 370.93; 370.93; 370.94; 370.97; 370.98; 371.01; 371.1; 371.11; 371.11; 371.12 | 0; 0; 0.57; 0.57; 0.6; 0.6; 0.6; 0.6; 0.63; 0.67; 1.71; 1.71; 1.71; 1.72; 1.72; 1.75; 1.77; 1.79; 1.88; 1.89; 1.89; 1.9 | 0.038; 0.037; 0.028; 0.028; 0.028; 0.028; 0.028; 0.028; 0.027; 0.027; 0.016; 0.016; 0.016; 0.016; 0.016; 0.016; 0.016; 0.015; 0.015; 0.015; 0.015; 0.015 | 0.664; 0.664; 0.602; 0.602; 0.621; 0.621; 0.621; 0.621; 0.602; 0.602; 0.624; 0.624; 0.624; 0.624; 0.605; 0.605; 0.605; 0.605; 0.625; 0.625; 0.625; 0.625 | 400.64 | 5.62991e-09 | FALSE |
| PFDS | s(Year) + s(Moisture); s(Year) + Lipid; s(Year) + s(Lipid); s(Year) + Lipid + d15N + d13CS; s(Year) + s(Lipid) + d15N + d13CS; s(Year) + Lipid + s(d15N) + d13CS; s(Year) + s(Lipid) + s(d15N) + d13CS; s(Year) + Lipid + d15N + s(d13CS); s(Year) + s(Lipid) + d15N + s(d13CS); s(Year) + Lipid + s(d15N) + s(d13CS); s(Year) + s(Lipid) + s(d15N) + s(d13CS); s(Year) + s(Moisture) + Lipid + d15N + d13CS; s(Year) + s(Moisture) + s(Lipid) + d15N + d13CS; s(Year) + s(Moisture) + s(Lipid) + d15N + s(d13CS); s(Year) + s(Moisture) + Lipid + d15N + s(d13CS); s(Year) + Lipid + d15N; s(Year) + s(Lipid) + d15N; s(Year) + s(Moisture) + d15N; s(Year) + s(Moisture) + s(d15N); s(Year) + s(Moisture) + Lipid; s(Year) + s(Moisture) + s(Lipid); s(Year) + s(Moisture) + Lipid + d15N; s(Year) + s(Moisture) + s(Lipid) + d15N | 109.69; 109.9; 109.91; 110.73; 110.74; 110.75; 110.75; 110.77; 110.79; 110.81; 110.82; 110.86; 110.86; 110.86; 110.86; 110.9; 110.92; 110.97; 110.97; 111.07; 111.07; 111.41; 111.41 | 0; 0.21; 0.22; 1.03; 1.04; 1.05; 1.05; 1.08; 1.1; 1.12; 1.13; 1.17; 1.17; 1.17; 1.17; 1.21; 1.23; 1.28; 1.28; 1.38; 1.38; 1.71; 1.71 | 0.052; 0.047; 0.046; 0.031; 0.031; 0.031; 0.031; 0.03; 0.03; 0.03; 0.03; 0.029; 0.029; 0.029; 0.029; 0.028; 0.028; 0.027; 0.027; 0.026; 0.026; 0.022; 0.022 | 0.869; 0.818; 0.818; 0.824; 0.824; 0.824; 0.824; 0.824; 0.824; 0.824; 0.824; 0.905; 0.905; 0.905; 0.905; 0.824; 0.824; 0.88; 0.88; 0.874; 0.874; 0.892; 0.892 | 162.6 | 1.682045e-13 | FALSE |
| ∑_13_PFCAs | s(Year) + Lipid; s(Year) + s(Lipid); s(Year) + Lipid + d15N; s(Year) + s(Lipid) + d15N; s(Year) + Moisture + Lipid; s(Year) + Moisture + s(Lipid); s(Year) + s(Moisture) + Lipid; s(Year) + s(Moisture) + s(Lipid); s(Year) + s(Moisture) + Lipid + d15N; s(Year) + s(Moisture) + s(Lipid) + d15N; s(Year) + Moisture + Lipid + d15N | 266.88; 266.88; 267.65; 267.68; 267.72; 267.73; 267.88; 267.88; 268.08; 268.13; 268.86 | 0; 0.01; 0.78; 0.8; 0.84; 0.85; 1.01; 1.01; 1.2; 1.25; 1.99 | 0.093; 0.093; 0.063; 0.062; 0.061; 0.061; 0.056; 0.056; 0.051; 0.05; 0.035 | 0.937; 0.937; 0.939; 0.939; 0.94; 0.94; 0.95; 0.95; 0.954; 0.954; 0.942 | 368.12 | 9.624472e-24 | FALSE |
| ∑_4_PFSAs | s(Year) + Lipid + s(d15N) + d13CS; s(Year) + s(Lipid) + s(d15N) + d13CS; s(Year) + s(Lipid) + d13CS; s(Year) + Lipid + d13CS; s(Year) + Lipid + s(d13CS); s(Year) + s(Lipid) + s(d13CS); s(Year) + Moisture + Lipid + s(d15N); s(Year) + s(Moisture) + Lipid + s(d15N); s(Year) + s(Moisture) + s(Lipid) + s(d15N); s(Year) + Moisture + s(Lipid) + s(d15N); s(Year) + Lipid + s(d15N); s(Year) + s(Lipid) + s(d15N); s(Year) + Lipid + d15N + d13CS; s(Year) + Lipid + d15N + s(d13CS); s(Year) + s(Lipid) + d15N + d13CS; s(Year) + s(Lipid) + d15N + s(d13CS); Year + Lipid + s(d15N) + d13CS; Year + Lipid + s(d15N) + s(d13CS); Year + s(Lipid) + s(d15N) + d13CS; Year + s(Lipid) + s(d15N) + s(d13CS) | 371.44; 371.44; 371.82; 371.82; 371.88; 371.92; 371.96; 371.97; 371.97; 371.97; 372.72; 372.72; 372.76; 372.79; 372.8; 372.83; 373.33; 373.33; 373.33; 373.33 | 0; 0; 0.38; 0.38; 0.44; 0.48; 0.52; 0.53; 0.53; 0.53; 1.28; 1.28; 1.32; 1.35; 1.36; 1.39; 1.89; 1.89; 1.89; 1.89 | 0.041; 0.041; 0.034; 0.034; 0.033; 0.032; 0.032; 0.031; 0.031; 0.031; 0.022; 0.022; 0.021; 0.021; 0.021; 0.02; 0.016; 0.016; 0.016; 0.016 | 0.695; 0.695; 0.634; 0.634; 0.634; 0.634; 0.779; 0.779; 0.779; 0.779; 0.732; 0.732; 0.638; 0.638; 0.638; 0.638; 0.637; 0.637; 0.637; 0.637 | 405.94 | 1.320407e-09 | FALSE |
| ∑_17_PFASs | s(Year) + Lipid; s(Year) + s(Lipid); s(Year) + Moisture + Lipid; s(Year) + s(Moisture) + Lipid; s(Year) + Moisture + s(Lipid); s(Year) + s(Moisture) + s(Lipid); s(Year) + Lipid + s(d15N) + d13CS; s(Year) + Lipid + s(d15N) + s(d13CS); s(Year) + s(Lipid) + s(d15N) + s(d13CS); s(Year) + s(Lipid) + s(d15N) + d13CS; s(Year) + Lipid + s(d15N); s(Year) + s(Lipid) + s(d15N); s(Year) + s(Lipid) + d13CS; s(Year) + Lipid + d13CS; s(Year) + Lipid + s(d13CS); s(Year) + s(Lipid) + s(d13CS) | 381.61; 381.63; 382.38; 382.41; 382.41; 382.42; 382.55; 382.55; 382.55; 382.55; 382.72; 382.72; 383.31; 383.32; 383.37; 383.38 | 0; 0.02; 0.78; 0.8; 0.8; 0.81; 0.94; 0.94; 0.94; 0.94; 1.11; 1.11; 1.7; 1.71; 1.76; 1.78 | 0.049; 0.049; 0.033; 0.033; 0.033; 0.033; 0.031; 0.031; 0.031; 0.031; 0.028; 0.028; 0.021; 0.021; 0.02; 0.02 | 0.788; 0.788; 0.792; 0.792; 0.792; 0.792; 0.836; 0.836; 0.836; 0.836; 0.836; 0.836; 0.786; 0.786; 0.786; 0.786 | 439.37 | 1.411112e-14 | FALSE |

*“Null in top” indicates whether the null model is included among the top models (∆AICc < 2).

4. **Method for detecting significant trends using derivatives from a GAM model**

**Fitting the GAM (Generalized Additive Model)**

The gam() function from the mgcv package was used to fit a GAM model [6]. The model is specified using the formula: compound ~ s(Year), where *compound* is the variable of interest (compound concentration) and *Year* is the explanatory variable. Model fitting was performed using the REML (Restricted Maximum Likelihood) method, which is commonly used to estimate spline parameters in additive models.

**Calculation of derivatives and confidence intervals**

From the fitted GAM, derivatives of the smooth function (i.e., the trend of the variable over time) are calculated using the derivatives() function from the gratia package [7]. These derivatives allow assessment of the slope of the curve and thus the identification of portions of trends where it is significantly increasing or decreasing. A 95% confidence interval was also computed for each derivative.

**Trend classification**

Based on the confidence intervals of the derivatives, trends are classified as follows:

- “Significantly increasing” if the lower bound of the confidence interval is greater than zero,
- “Significantly decreasing” if the upper bound of the confidence interval is less than zero,
- “Neutral” if the confidence interval includes zero, indicating a non-significant trend.

**Graphical illustration - Derivatives over time**

**Figure S2.** Temporal derivatives (f’(x)) of lipid-standardized PFOS (the compound with the highest relative contribution) concentrations and identification of significant trends. This figure shows the rate of change in PFOS concentrations over time, after correction for lipid content. The blue line represents the estimated temporal trend, surrounded by its 95% confidence interval. Sections where the confidence interval lies entirely below zero are shown in green, indicating a significant decreasing trend. Conversely, sections where the confidence interval lies entirely above zero are shown in red, indicating a significant increasing trend.

**Estimation of the maximum and its confidence interval**

The predict() function from the mgcv package was used to estimate concentration values for each year by generating a sequence of years from the first to the last available in the dataset [6]. The peak of the smoothed curve was detected, and the corresponding year was identified. A 95% confidence interval for the year of the peak concentration was also estimated.

**Creation of trend segments**

Significantly increasing or decreasing segments were extracted and assigned a unique identifier in R. Neutral segments (neither increasing nor decreasing) were excluded, allowing a focus solely on periods of significant variation.

**Adding differentiated trend segments to a plot (ggplot2)**

These differentiated trend segments can be added to time series plots using the geom_line() function from the ggplot2 package [8]. This visualization clearly highlights periods of significant change, facilitating the interpretation of the temporal dynamics of the studied compound.

5. **Temporal trends in PFAS concentrations**

**Table S4.** Year of maximum concentrations [year–95% CI - year+95% CI] and associated concentrations [concentration–95% CI - concentration+95% CI], periods of significant increase and decrease (and associated concentration ranges), and ecological half-lives of PFAS, corrected for lipid content and for both lipid content and stable nitrogen isotope ratios (δ^15^N).

| Compound ^a^ | Lipid correction | | | | | Lipid and δ^15^N correction | | | | |
| --- | --- | --- | --- | --- | --- | --- | --- | --- | --- | --- |
|  | Year of maximum | Concentration at maximum (ng/g ww) | Increasing periods | Decreasing periods | T_1/2_ (years) | Year of maximum | Concentration at maximum (ng/g ww) | Increasing periods | Decreasing periods | T_1/2_ (years) |
| PFHxS (C6) | 1999 [1994 - 2004] | 0.68 [0.60 - 0.75] | 1988 - 1996 (0.46 - 0.65 ng/g ww) | 2002 - 2010 (0.65 - 0.51 ng/g ww) | 41 | 1999 [1994 - 2004] | 0.69 [0.61 - 0.77] | 1988 - 1996 (0.47 - 0.66 ng/g ww) | 2002 - 2010 (0.66 - 0.51 ng/g ww) | 38 |
| PFOS (C8) | 1994 [1987 - 2001] | 100.33 [89.80 - 110.86] | 1976 - 1986 (61.99 - 90.23 ng/g ww) | 1999 - 2005 (95.16 - 82.30 ng/g ww) | 54 | 1997 [1986 - 2006] | 101.95 [90.39 - 113.51] | 1981 - 1985 (82.57 - 89.22 ng/g ww) | 2002 - 2019 (97.60 - 62.62 ng/g ww) | 21 |
| PFDS (C10) | 1994 [1988 - 1999] | 5.95 [5.34 - 6.56] | 1975 - 1990 (2.58 - 5.68 ng/g ww) | 1998 - 2008 (5.63 - 2.80 ng/g ww) | 28 | 1997 [1990 - 2002] | 6.21 [5.43 - 6.99] | 1976 - 1990 (2.77 - 5.60 ng/g ww) | 2000 - 2010 (5.96 - 2.85 ng/g ww) | 17 |
| ∑_4_PFSAs | 1994 [1987 - 2001] | 107.32 [96.17 - 118.46] | 1976 - 1986 (64.47 - 95.90 ng/g ww) | 1998 - 2005 (102.65 - 86.09 ng/g ww) | 52 | 1996 [1986 - 2005] | 105.67 [95.01 - 116.33] | 1976 - 1988 (78.63 - 98.72 ng/g ww) | 2001 - 2016 (101.39 - 74.57 ng/g ww) | 27 |
| PFOA (C8) | 1999 [1994 - 2005] | 0.93 [0.83 - 1.04] | 1988 - 1996 (0.60 - 0.89 ng/g ww), 2017 - 2021 (0.63 - 0.74 ng/g ww) | 2003 - 2011 (0.88 - 0.63 ng/g ww) | 85 | 2000 [1996 - 2005] | 1.07 [0.96 - 1.19] | 1988 - 1998 (0.56 - 1.04 ng/g ww), 2018 - 2021 (0.56 - 0.63 ng/g ww) | 2003 - 2013 (1.04 - 0.56 ng/g ww) | 51 |
| PFNA (C9) | 2006 [2001 - 2011] | 11.75 [10.12 - 13.39] | 1980 - 1988 (5.03 - 8.37 ng/g ww), 2000 - 2004 (9.17 - 11.34 ng/g ww) | 2009 - 2014 (11.21 - 8.61 ng/g ww) | 36 | 2006 [2001 - 2010] | 13.03 [11.18 - 14.88] | 1982 - 1986 (5.68 - 7.50 ng/g ww), 1998 - 2003 (9.50 - 12.52 ng/g ww) | 2008 - 2015 (12.51 - 7.74 ng/g ww) | 24 |
| PFDA (C10) | ND | ND | 1969 - 2024 (2.62 - 6.89 ng/g ww) | ND | ND | 2009 [2000 - 2018] | 6.24 [5.64 - 6.84] | 1981 - 2004 (3.46 - 6.03 ng/g ww) | ND | 32 |
| PFUdA (C11) | 2007 [2002 - 2012] | 19.16 [17.16 - 21.17] | 1977 - 1985 (8.77 - 12.73 ng/g ww), 1998 - 2004 (14.66 - 18.51 ng/g ww) | 2010 - 2013 (18.32 - 16.59 ng/g ww) | 34 | 2006 [2001 - 2010] | 20.67 [18.41 - 22.92] | 1980 - 1984 (10.32 – 12.08 ng/g ww), 1997 - 2003 (14.82 - 19.98 ng/g ww) | 2008 - 2015 (19.93 - 14.90 ng/g ww) | 25 |
| PFDoA (C12) | 2007 [2001 - 2014] | 4.80 [4.32 - 5.29] | 1976 - 1985 (2.38 - 3.78 ng/g ww), 1999 - 2003 (4.01 - 4.60 ng/g ww) | ND | 47 | 2006 [1997 - 2016] | 4.67 [4.24 - 5.10] | 1976 - 1989 (2.77 - 3.80 ng/g ww), 1998 - 2000 (4.32 - 4.42 ng/g ww) | ND | 34 |
| PFTrDA (C13) | 2005 [2002 - 2009] | 13.92 [12.49 - 15.35] | 1977 - 1984 (6.14 - 9.10 ng/g ww), 1997 - 2003 (10.00 - 13.56 ng/g ww) | 1969 - 1971 (7.19 - 6.54 ng/g ww), 2007 - 2014 (13.65 - 9.12 ng/g ww) | 35 | 2005 [2002 - 2008] | 14.94 [13.45 - 16.43] | 1978 - 1984 (6.65 - 8.67 ng/g ww), 1996 - 2003 (9.83 - 14.62 ng/g ww) | 2006 - 2014 (14.70 - 8.70 ng/g ww) | 27 |
| PFTeDA (C14) | 2004 [1997 - 2009] | 2.97 [2.66 - 3.27] | 1976 - 1985 (1.47 - 2.17 ng/g ww), 1996 - 2000 (2.60 - 2.81 ng/g ww) | 2007 - 2012 (2.86 - 2.49 ng/g ww) | 52 | 2004 [1998 - 2008] | 3.14 [2.83 - 3.45] | 1978 - 1985 (1.59 - 2.08 ng/g ww), 1993 - 2001 (2.46 - 3.04 ng/g ww) | 2006 - 2013 (3.04 - 2.34 ng/g ww) | 35 |
| ∑_13_PFCAs | 2006 [2001 - 2011] | 58.07 [52.05 - 64.10] | 1977 - 1985 (27.14 - 40.04 ng/g ww), 1998 - 2003 (46.81- 55.95 ng/g ww) | 2009 - 2013 (56.32 - 49.18 ng/g ww) | 41 | 2006 [2002 - 2010] | 62.40 [55.93 - 68.87] | 1979 - 1985 (29.96 - 38.47 ng/g ww), 1997 - 2003 (46.30 - 60.45 ng/g ww) | 2008 - 2015 (60.69 - 45.30 ng/g ww) | 31 |
| ∑_17_PFASs | 1996 [1988 - 2008] | 151.56 [137.72 - 165.39] | 1975 - 1990 (92.17 - 144.54 ng/g ww) | ND | 60 | 1998 [1989 - 2008] | 154.00 [140.75 - 167.25] | 1976 - 1991 (106.98 - 145.88 ng/g ww) | 2005 - 2018 (147.52 - 117.42 ng/g ww) | 36 |

Abbreviation: ND, not detected.

^a^ PFBS, PFBA, PFPeA, PFHxA, PFHpA, PFHxDA, and PFODA did not yield data or sufficient data above detection limits.

**Figure S3.** Temporal trends in PFAS concentrations (ng/g ww) in northern gannet (*Morus bassanus*) eggs from Bonaventure Island: PFOA, PFNA, PFDA, PFUdA, PFDoA, PFTrDA, PFTeDA, PFHxS, ∑_4_PFSAs and PFDS. Two trend curves are shown: in purple, concentrations corrected for both lipid content and stable nitrogen isotope ratios (δ^15^N); in blue, concentrations corrected for lipid content only. Likewise, inflection points are displayed with (purple) and without (blue) δ^15^N correction. Colored segments highlight significant increases (red) and decreases (green). Colored bars represent periods of voluntary production phase-out and regulation in North America: PFOS phase-out (2000–2003; dark red), PFOA and long-chain PFCAs (LC-PFCAs) phase-out (2010–2015; orange), and regulatory period (2002–2024; green).

8. **Method for comparing relative contributions (%) before and after the year of maximum**

**Statistical analysis steps**

For each compound studied, the proportions are divided into two groups: before and after the peak.

**Normality test**

To determine if the data followed a normal distribution, a Shapiro-Wilk test was independently applied to both groups (before and after). If both p-values were > 0.05, normality was assumed for both groups.

**Test for homogeneity of variances**

If normality is met, a Levene's test is then used to check if the variances of the two groups can be considered equal. A p-value > 0.05 suggests equality of variances.

**Choice of comparison**

Based on the results from the previous steps, an appropriate test is selected to compare the two groups:

- If normality + equality of variances: classic Student's t-test;
- If normality but unequal variances: Welch's t-test, which does not assume equal variances;
- If non-normality in at least one group: non-parametric Wilcoxon test.

**Results of the statistical comparison of PFAS relative contributions before and after the peak year**

**Table S5.** Statistical comparison of the relative contributions of individual PFAS compounds before and after their peak year. For each compound, the table reports the year of maximum concentration, results of Shapiro-Wilk tests for normality before and after the peak (p-values), the Levene’s test for equality of variances, the statistical test used to compare the two periods, and the corresponding p-value. The table also includes the median relative contributions (%) of each compound before and after the maximum year.

| Compound | Year of maximum | Normality before the maximum (Shapiro test, p-value) | Normality after the maximum (Shapiro test, p-value) | Equality of variances (Levene’s test, p-value) | Test | Comparison before vs after  (p-value) | Median group before (%) | Median group after (%) |
| --- | --- | --- | --- | --- | --- | --- | --- | --- |
| PFOS (C8) | 1994 | 0.2063 | 0.4532 | 0.4199 | t-test | <0.001 | 66.9 | 60.5 |
| PFHxS (C6) | 1999 | 1e-04 | 0.7155 | 0.0598 | Wilcoxon | 0.032 | 0.36 | 0.41 |
| PFDS (C10) | 1994 | 7e-04 | 0.0481 | 0.0016 | Wilcoxon | 0.57 | 2.54 | 2.48 |
| PFOA (C8) | 1999 | 0 | 0.0438 | 0.6384 | Wilcoxon | 0.086 | 0.49 | 0.51 |
| PFNA (C9) | 2006 | 0.3761 | 0.0407 | 0.6993 | Wilcoxon | 0.0014 | 5.58 | 6.60 |
| PFUdA (C11) | 2007 | 0.0209 | 0.0153 | 0.1031 | Wilcoxon | <0.001 | 9.79 | 12.4 |
| PFDoA (C12) | 2007 | 0.1656 | 0.8037 | 0.0088 | Welch t-test | <0.001 | 2.79 | 3.42 |
| PFTrDA (C13) | 2005 | 1e-04 | 0.8053 | 0.1385 | Wilcoxon | 0.054 | 7.01 | 7.60 |
| PFTeDA (C14) | 2004 | 0.0604 | 0.0307 | 0.8314 | Wilcoxon | <0.001 | 1.64 | 1.96 |

10. **PFAS regulations**

**Table S6.** PFAS regulations in the studied region and internationally: regulations/limits, responsible organization or country, effective date, targeted PFAS substances, and references.

| Regulations/ Limits | Organization/ Country | Effective Date | Targeted PFAS | References |
| --- | --- | --- | --- | --- |
| SNUR (Significant New Use Rule) - Two provisions requiring manufacturers to notify the EPA before manufacturing or importing 13, then 75 PFAS chemical substances | USA | 2002 | A list of 75 PFAS substances | [9] [10] |
| Voluntary phase-out of PFOS production by 3M™ | USA | 2000–2003 | PFOS | [9] [11] |
| Risk Management Strategy for PFOS, its salts and precursors | Canada | 2006 | PFOS, its salts and precursors | [9] [12] |
| PFOA Stewardship Program - The U.S. Environmental Protection Agency invited eight major PFAS producers to participate in a voluntary program aiming for a 95% reduction in facility emissions and product content of PFOA by 2010, and complete elimination by 2015 | USA | 2006 | PFOA | [9] [13] |
| Directive 2006/122/EC on marketing and use restrictions of PFOS | European Union | 2006 | PFOS | [9] [14] |
| Chapter 173-333, Washington Administrative Code, restricting the use and environmental release of PFOS | Washington (USA) | 2006 | PFOS | [15] |
| Substance prohibition summary for Perfluorooctane Sulfonate, its salts and precursors (PFOS) | Canada | 2008 | PFOS | [16] |
| Stockholm Convention: listing of perfluorooctane sulfonic acid (PFOS), its salts, and perfluorooctane sulfonyl fluoride in Annex B of the Persistent Organic Pollutants (POPs) treaty | International | 2009 | PFOS | [17] [18] |
| Canadian Environmental Protection Act (CEPA) - Schedule 3 - Export Control List (Restricted Substances) | Canada | 2012 | PFOS | [19] |
| Maximum Contaminant Level (MCL) for PFNA | New Jersey (USA) | 2015 | PFNA | [9] [20] |
| Perfluorocarboxylic Acids and Their Precursors: Environmental Performance Agreement Overview - An environmental performance agreement concerning perfluorocarboxylic acids (PFCAs) and their precursors was negotiated between Environment and Climate Change Canada, Health Canada, and companies such as Arkema Canada, Asahi Glass Company, Clariant Canada, and E.I. du Pont Canada. This agreement, effective since 2015, led to the elimination of the production of PFOA, long-chain PFCAs, and their precursors. | Canada | 2015 | PFOA, long-chain PFCAs, and their precursors | [21] [22] |
| Ban on the use of PFOS‑based fume suppressants in chromium plating operations | USA | 2015 | PFOS | [23] |
| Ban on the manufacture, use, sale, and import of PFOA and certain long-chain PFCAs | Canada | 2016 | PFOA, certain long-chain PFCAs and their salts | [24] [25] |
| Lifetime Health Advisory: threshold of 70 ppt in drinking water | USA | 2016 | PFOS, PFOA | [9] [26] |
| Adoption of amendments to 6 NYCRR Part 597 adding PFOA and PFOS to the New York State list of hazardous substances | New York (USA) | 2017 | PFOS, PFOA | [27] |
| Commission Regulation (EU) 2017/1000 - Amendment of Annex XVII to REACH concerning PFOA, its salts and related substances | European Union | 2017 | PFOA | [9] [28] |
| Canadian Environmental Protection Act (CEPA) - Schedule 3: Export Control List (substances subject to notification or consent) | Canada | 2018 | PFOS, its salts and compounds, PFOA, its salts and precursors | [29] |
| RCW 70A.222.070: Restricts intentionally added PFAS in food packaging | Washington (USA) | 2018 | PFAS | [30] [31] |
| RCW 70A.400: PFAS in firefighting - Restrictions on Class B firefighting foam and firefighting personal protective equipment | Washington (USA) | 2018 | PFAS | [30] [32] |
| Wisconsin Act 101 - Restrictions on PFAS‑containing firefighting foam use and disposal | Wisconsin (USA) | 2019 | PFAS | [9] [33] |
| Senate Bill No. 1044: prohibits Class B firefighting foam containing intentionally added PFAS (with limited exceptions) and requires disclosure of PFAS in firefighter protective equipment | California (USA) | 2020 | PFAS | [9] [34] |
| House Bill 19‑1279: bans the use and sale of certain PFAS in Class B firefighting foam and requires inventory reporting and collection programs | Colorado (USA) | 2019 | PFAS | [9] [35] |
| PFAS Action Plan: EPA finalized enforceable MCLs for PFOA and PFOS in drinking water and designated them as hazardous substances under CERCLA to strengthen cleanup and accountability | USA | 2024 | PFOS, PFOA | [9] [36] |
| Stockholm Convention: listing of perfluorooctanoic acid (PFOA), its salts, and related compounds in Annex A of the Persistent Organic Pollutants (POPs) treaty | International | 2019 | PFOA | [17] [37] |
| Maine Public Law Chapter 277 (LD 1433): Toxic chemicals in packaging | Maine (USA) | 2019 | PFAS | [9] [38] |
| DEQ-7: Water Quality Standards | Montana (USA) | 2019 | PFOS, PFOA | [9] [39] |
| Ohio PFAS Action Plan for Drinking Water: established state action levels and response protocols for PFAS in public water systems | Ohio (USA) | 2019 | PFAS | [9] [40] |
| Proposed ban on use of PFAS firefighting foam for training | Ohio (USA) | 2019 | PFAS | [9] [41] |
| Addition of certain PFAS to the Toxics Release Inventory (TRI) list under the National Defense Authorization Act (NDAA) | USA | 2019 | PFPrA, PFHxA and its salts, PFDA and its salts, PFHxS-Na, 6:2 FTS and its salts, TFSI, LiTFSI, and other PFAS | [9] [42] |
| Act No. 21 (S.49): requires public water systems to monitor PFAS levels and sets regulatory limits and control requirements for PFAS in drinking water and surface waters | Vermont (USA) | 2019 | PFAS | [9] [43] [44] |
| SB 121: establishes regulatory requirements for PFAS pollutants and firefighting substances, including monitoring and control | Alaska (USA) | 2021 | PFAS | [9] [45] |
| Establishment of Maximum Contaminant Levels (MCLs) for PFAS in drinking water | New Hampshire (USA) | 2019 | PFOS, PFOA, PFNA, PFHxS | [9] [46] |
| MCLs for PFAS in drinking water: legal maximum contaminant levels set for 7 PFAS compounds | Michigan (USA) | 2020 | PFNA, PFOA, PFHxA, PFOS, PFHxS, PFBS, HFPO-DA | [9] [47] |
| Per- and polyfluoroalkyl substances (PFAS) in pesticide and other packaging | USA | 2021 | PFAS | [48] |
| Stockholm Convention: listing of perfluorohexane sulfonic acid (PFHxS), its salts, and PFHxS‑related compounds in Annex A of the Persistent Organic Pollutants (POPs) treaty | International | 2022 | PFHxS | [17] [49] |
| New Laws for Household Cleaning, Personal Care, Cosmetics, Food Packaging, and Children's Products Help Prevent Public Exposure | New York (USA) | 2022 | PFAS | [50] |
| An Act to amend the laws relating to the prevention of perfluoroalkyl and polyfluoroalkyl substances pollution and to provide additional funding | Maine (USA) | 2023 | PFAS | [51] |
| DEC Releases Final Ambient Water Quality Guidance Values for PFOA and PFOS | New York (USA) | 2023 | PFOS, PFOA | [52] |

**References**

[1] Burnham, K. P., and Anderson, D. R. (Eds.). (2002). *Model selection and multimodel inference*. New York, NY: Springer New York. https://doi.org/10.1007/b97636

[2] Pala, N., Vorkamp, K., Bossi, R., Bignert, A., Traversa, G., Fugazza, D., Ancora, S., Ademollo, N., Baroni, D., and Corsolini, S. (2025). Temporal trends of persistent organic pollutants (POPs) and perfluoroalkyl substances (PFAS) in Adèlie penguin (*Pygoscelis adeliae*) eggs from the Ross Sea (Antarctica), including their relationship with climate parameters. *Environmental Pollution*, *373*, 126130. https://doi.org/10.1016/j.envpol.2025.126130

[3] Bustnes, J. O., Bårdsen, B.-J., Herzke, D., Bangjord, G., Bourgeon, S., Fritsch, C., and Eulaers, I. (2022). Temporal trends of organochlorine and perfluorinated contaminants in a terrestrial raptor in northern europe over 34 years (1986–2019). *Environmental Toxicology and Chemistry*, *41*(6), 1508‑1519. https://doi.org/10.1002/etc.5331

[4] Hashizume, N., Inoue, Y., Suzuki, Y., Murakami, H., Sumi, S., Ishibashi, T., and Yoshida, T. (2017). Comparison of laboratory-derived biomagnification factors for hexachlorobenzene in common carp conducted under 9 test conditions. *Environmental Toxicology and Chemistry*, *37*(4), 1032‑1039. https://doi.org/10.1002/etc.4030

[5] Miranda, D. A., Benskin, J. P., Awad, R., Lepoint, G., Leonel, J., and Hatje, V. (2021). Bioaccumulation of per- and polyfluoroalkyl substances (PFASs) in a tropical estuarine food web. *Science of The Total Environment*, *754*, 142146. https://doi.org/10.1016/j.scitotenv.2020.142146

[6] Wood, S. N. (2011). Fast stable restricted maximum likelihood and marginal likelihood estimation of semiparametric generalized linear models. *Journal of the Royal Statistical Society Series B: Statistical Methodology*, *73*(1), 3‑36. <https://doi.org/10.1111/j.1467-9868.2010.00749.x>

[7] Simpson, G. L. (2024). gratia: An R package for exploring generalized additive models. *Journal of Open Source Software*, *9*(104), 6962. <https://doi.org/10.21105/joss.06962>

[8] Wickham, H. (2016). *ggplot2: Elegant Graphics for Data Analysis*. Springer-Verlag New York. ISBN 978-3-319-24277-4. <https://ggplot2.tidyverse.org>

[9] Brennan, N. M., Evans, A. T., Fritz, M. K., Peak, S. A., and von Holst, H. E. (2021). Trends in the regulation of per- and polyfluoroalkyl substances (PFAS): A scoping review. *International Journal of Environmental Research and Public Health*, *18*(20), 10900. https://doi.org/10.3390/ijerph182010900

[10] U.S. Environmental Protection Agency (EPA). (2002). *Perfluoroalkyl sulfonates; significant new use rule*. *Federal Register, 67*(236), 72854–72867. Retrieved April 16, 2025, from [https://www.govinfo.gov/content/pkg/FR-2002-12-09/pdf/02-31011.pdf](https://www.govinfo.gov/content/pkg/FR-2002-12-09/pdf/02-31011.pdf?utm_source=chatgpt.com)

[11] U.S. Environmental Protection Agency (EPA). (2000). *EPA and 3M announce phase out of PFOS.* Retrieved April 16, 2025, from <https://www.epa.gov/archive/epapages/newsroom_archive/newsreleases/33aa946e6cb11f35852568e1005246b4.html>

[12] Government of Canada. (2006). *Risk management strategy for perfluorooctane sulfonate and its salts and precursors.* Retrieved April 16, 2025, from https://www.canada.ca/en/environment-climate-change/services/canadian-environmental-protection-act-registry/publications/risk-management-strategy-perfluorooctane-sulfonate.html

[13] U.S. Environmental Protection Agency (EPA). (2025). *Fact sheet: 2010/2015 PFOA Stewardship Program.* Retrieved April 16, 2025, from <https://www.epa.gov/assessing-and-managing-chemicals-under-tsca/fact-sheet-20102015-pfoa-stewardship-program>

[14] European Parliament and Council of the European Union. (2006). Directive 2006/122/EC of the European Parliament and of the Council of 12 December 2006 amending Council Directive 76/769/EEC on restrictions on the marketing and use of certain dangerous substances and preparations (perfluorooctane sulfonates) (Text with EEA relevance). Official Journal of the European Union, L 372, 32–34. <http://data.europa.eu/eli/dir/2006/122/oj>

[15] Washington State Legislature. (2006). *Chapter 173-333, Washington Administrative Code.* Retrieved April 16, 2025, from https://app.leg.wa.gov/WAC/default.aspx?cite=173-333

[16] Government of Canada. (2025a). *Substance prohibition summary for Perfluorooctane Sulfonate, its salts and precursors (PFOS).* Retrieved December 31, 2025, from https://www.canada.ca/en/environment-climate-change/services/management-toxic-substances/prohibition-certain-toxic-substances-regulations-2025/perfluorooctane-sulfonate.html

[17] Stockholm Convention. (2001). *Stockholm Convention on Persistent Organic Pollutants (POPs).* Retrieved April 16, 2025, from <https://www.pops.int/TheConvention/Overview/TextoftheConvention>

[18] United Nations Environment Programme (UNEP). (2009). *Decision SC‑4/17: Listing of perfluorooctane sulfonic acid, its salts and perfluorooctane sulfonyl fluoride in Annex B of the Stockholm Convention on Persistent Organic Pollutants.* https://chm.pops.int

[19] Government of Canada. (2012). *Export Control List, Schedule 3.* Retrieved April 16, 2025, from https://www.canada.ca/en/environment-climate-change/services/canadian-environmental-protection-act-registry/historical/export-control-list-september-12-2012.html

[20] New Jersey Drinking Water Quality Institute Health Effects Subcommittee. (2015). *Health-based maximum contaminant level support document: Perfluorononanoic Acid (PFNA).* Retrieved April 16, 2025, from https://www.nj.gov/dep/watersupply/pdf/pfna-health-effects.pdf

[21] Environment and Climate Change Canada. (2006). *Action plan for the assessment and management of perfluorocarboxylic acids (PFCAs) and their precursors [PDF, archived]. Archived from the original on August 24, 2017.* Retrieved April 16, 2025, from https://wayback.archive-it.org/7084/20170824184903/http://www2.ec.gc.ca/epe-epa/AE06B51E-7BB7-4C92-A4B5-6027E9195712/AFPC_EPE-FR.pdf

[22] Government of Canada. (2018). *Perfluorocarboxylic acids and their precursors: environmental performance agreement overview.* Retrieved April 16, 2025, from https://www.canada.ca/en/environment-climate-change/services/environmental-performance-agreements/list/perfluorocarboxylic-acids-overview.html

[23] California Air Resources Board. (2023). *Chrome Plating – Fume Suppressant Information.* Retrieved April 16, 2025, from <https://ww2.arb.ca.gov/resources/documents/chrome-plating-fume-suppressant-information>

[24] Government of Canada. (2025b). *Toxic substances list: perfluorooctanoic acid (PFOA), its salts and precursors.* Retrieved December 31, 2025, from <https://www.canada.ca/en/environment-climate-change/services/management-toxic-substances/list-canadian-environmental-protection-act/perfluorooctanoic-acid.html>

[25] Government of Canada. (2025c). *Toxic substances list: long-chain perfluorocarboxylic acids (LC-PFCAs), their salts and precursors.* Retrieved December 31, 2025, from https://www.canada.ca/en/environment-climate-change/services/management-toxic-substances/list-canadian-environmental-protection-act/long-chain-perfluorocarboxylic-acids.html

[26] U.S. Environmental Protection Agency (EPA). (2016). *Lifetime health advisories and health effects support documents for perfluorooctanoic acid (PFOA) and perfluorooctane sulfonate (PFOS)* [PDF]. Retrieved April 16, 2025, from https://www.epa.gov/sites/default/files/2016-05/documents/pfoa_pfos_prepub_508.pdf

[27] New York State Department of Environmental Conservation. (2017). *Adoption Of Final Rule: 6 NYCRR Part 597.* Retrieved April 16, 2025, from https://dec.ny.gov/regulatory/regulations/adoption-of-final-rule-6-nycrr-part-597

[28] European Commission. (2017). Commission Regulation (EU) 2017/1000 of 13 June 2017 amending Annex XVII to Regulation (EC) No 1907/2006 of the European Parliament and of the Council concerning the Registration, Evaluation, Authorisation and Restriction of Chemicals (REACH) as regards perfluorooctanoic acid (PFOA), its salts and PFOA-related substances. *Official Journal of the European Union, L 150*, 14–18. https://eur-lex.europa.eu/legal-content/EN/TXT/PDF/?uri=CELEX:32017R1000

[29] Government of Canada (2025d). *Export Control List.* Retrieved April 16, 2025, from https://www.canada.ca/en/environment-climate-change/services/canadian-environmental-protection-act-registry/substances-list/export-control-list.html

[30] Washington State Department of Ecology. (n.d.). *Per- and polyfluoroalkyl substances (PFAS).* Retrieved April 16, 2025, from https://ecology.wa.gov/waste-toxics/reducing-toxic-chemicals/addressing-priority-toxic-chemicals/pfas

[31] Washington State Legislature. (n.d.). *Chapter 70A.222 RCW: Packages containing metals and toxic chemicals.* Retrieved April 16, 2025, from https://app.leg.wa.gov/rcw/default.aspx?cite=70A.222

[32] Washington State Legislature. (n.d.). *Chapter 70A.400 RCW: Firefighting agents and equipment—Toxic chemical use.* Retrieved April 16, 2025, from https://app.leg.wa.gov/rcw/default.aspx?cite=70A.400

[33] State of Wisconsin. (2020). *2019 Wisconsin Act 101: An act to create 299.48 of the statutes; relating to regulating firefighting foam that contains certain contaminants and granting rule‑making authority.* Madison, WI: Wisconsin Legislature.

[34] State of California. (2020). *Senate Bill No. 1044: Firefighting Equipment and Foam (Chapter 308, Statutes of 2020).* Sacramento, CA: California Legislature. Retrieved April 16, 2025, from https://leginfo.legislature.ca.gov/faces/billTextClient.xhtml?bill_id=201920200SB1044

[35] Colorado General Assembly. (2019). *House Bill 19‑1279: Concerning the use of perfluoroalkyl and polyfluoroalkyl substances, and, in connection therewith, making an appropriation (Signed bill).* Retrieved April 16, 2025, from http://leg.colorado.gov/bill_files/61070/download

[36] U.S. Environmental Protection Agency (EPA). (2024a). *Key EPA actions to address PFAS.* Retrieved April 16, 2025, from https://www.epa.gov/pfas/key-epa-actions-address-pfas

[37] United Nations Environment Programme (UNEP). (2019). *Decision SC‑9/12: Listing of perfluorooctanoic acid (PFOA), its salts and related compounds in Annex A of the Stockholm Convention on Persistent Organic Pollutants.* https://chm.pops.int

[38] State of Maine. (2019). *An Act to Protect the Environment and Public Health by Further Reducing Toxic Chemicals in Packaging* (Public Law Chapter 277, 129th Legislature). Retrieved April 16, 2025, from https://www.mainelegislature.org/legis/bills/bills_129th/billtexts/HP104301.asp

[39] Montana Department of Environmental Quality. (2019). *Circular DEQ-7 Montana Numeric Water Quality Standards.* Retrieved April 16, 2025, from https://deq.mt.gov/files/Water/WQPB/Standards/PDF/DEQ7/DEQ-7.pdf

[40] Ohio Environmental Protection Agency and Ohio Department of Health. (2019). *Ohio Per- and Polyfluoroalkyl Substances (PFAS) Action Plan for Drinking Water.* Retrieved April 16, 2025, from https://butlertownship.com/wp-content/uploads/2022/02/Ohio-EPA-PFAS-Action-Plan.pdf

[41] Garbe, W. (2019). Bill would stop Ohio firefighters from training with PFAS-based foam. *Dayton Daily News.* Retrieved April 16, 2025, from https://www.daytondailynews.com/news/state--regional-govt--politics/bill-would-prohibit-ohio-firefighters-from-using-certain-foam-training/Y1EK2LtZfoNlbkwSYyCUEK/

[42] U.S. Environmental Protection Agency (EPA). (2024b). *Addition of certain per- and polyfluoroalkyl substances (PFAS) to the Toxics Release Inventory by the National Defense Authorization Act* (Section 7321, National Defense Authorization Act for Fiscal Year 2020). Retrieved April 16, 2025, from https://www.epa.gov/toxics-release-inventory-tri-program/addition-certain-pfas-tri-national-defense-authorization-act

[43] Vermont General Assembly. (2019). *Act No. 21 (S.49): An Act relating to the regulation of perfluoroalkyl and polyfluoroalkyl substances in drinking and surface waters.* Retrieved April 16, 2025, from https://legislature.vermont.gov/Documents/2020/Docs/ACTS/ACT021/ACT021%20As%20Enacted.pdf

[44] Vermont Department of Environmental Conservation. (2020). *Advanced notice on the regulation of PFAS as a class.* Retrieved April 16, 2025, from https://dec.vermont.gov/sites/dec/files/PFAS/20180814-PFAS-as-a-Class.pdf

[45] Alaska State Legislature. (2021). *Senate Bill No. 121: An Act relating to pollutants; relating to perfluoroalkyl and polyfluoroalkyl substances; relating to the duties of the Department of Environmental Conservation; relating to firefighting substances; relating to thermal remediation of perfluoroalkyl and polyfluoroalkyl substance contamination; and providing for an effective date* (SB 121). Retrieved April 16, 2025, from http://www.akleg.gov/basis/Bill/Text/32?Hsid=SB0121A

[46] New Hampshire Department of Environmental Services. (2019). *Technical Background Report for the June 2019 Proposed Maximum Contaminant Levels (MCLs) and Ambient Groundwater Quality Standards (AGQSs) for Perfluorooctane sulfonic Acid (PFOS), Perfluorooctanoic Acid (PFOA), Perfluorononanoic Acid (PFNA), and Perfluorohexane sulfonic Acid (PFHxS).* Retrieved April 16, 2025, from https://www.des.nh.gov/sites/g/files/ehbemt341/files/documents/r-wd-19-29.pdf

[47] Michigan Department of Environment, Great Lakes, and Energy (EGLE). (2020). *Maximum contaminant levels (MCLs) for PFAS in drinking water.* Retrieved April 16, 2025, from https://www.michigan.gov/pfasresponse/drinking-water/mcl

[48] U.S. Environmental Protection Agency (EPA). (2024c). *Per- and polyfluoroalkyl substances (PFAS) in pesticide and other packaging*. Retrieved April 16, 2025, from https://www.epa.gov/pesticides/pfas-packaging

[49] United Nations Environment Programme (UNEP). (2022). *Decision SC‑10/13: Initial indicative list of perfluorohexane sulfonic acid (PFHxS), its salts and PFHxS-related compounds in Annex A of the Stockholm Convention on Persistent Organic Pollutants.* https://chm.pops.int

[50] New York State Department of Environmental Conservation (DEC). (2022). DEC: *Several new requirements taking effect for 2023 to protect consumers from harmful chemicals*. Retrieved April 16, 2025, from https://dec.ny.gov/news/press-releases/2022/12/dec-several-new-requirements-taking-effect-for-2023-to-protect-consumers-from-harmful-chemicals

[51] State of Maine Legislature. (2023). *An Act to amend the laws relating to the prevention of perfluoroalkyl and polyfluoroalkyl substances pollution and to provide additional funding*. Augusta, ME, USA. Retrieved April 5, 2023, from https://www.mainelegislature.org/legis/bills/getPDF.asp?paper=SP0610&item=1&snum=131

[52] New York State Department of Environmental Conservation (DEC). (2023). *DEC Releases final ambient water quality guidance values for PFOA, PFOS, and 1,4-dioxane*. Retrieved April 16, 2025, from <https://dec.ny.gov/news/press-releases/2023/3/dec-releases-final-ambient-water-quality-guidance-values-for-pfoa-pfos-and-14-dioxane>
